# Investigation of USP30 inhibition to enhance Parkin-mediated mitophagy: tools and approaches

**DOI:** 10.1042/BCJ20210508

**Published:** 2021-12-06

**Authors:** Eliona Tsefou, Alison S. Walker, Emily H. Clark, Amy R. Hicks, Christin Luft, Kunitoshi Takeda, Toru Watanabe, Bianca Ramazio, James M. Staddon, Thomas Briston, Robin Ketteler

**Affiliations:** 1 MRC Laboratory for Molecular Cell Biology, University College London, London, U.K.; 2 UCL:Eisai Therapeutic Innovation Group, Translational Research Office, University College London, London, U.K.; 3 Neurology Innovation Centre, Hatfield Research Laboratories, Eisai Ltd., Hatfield, U.K.

**Keywords:** mitokeima, mitophagy, p-ser65-ubiquitin, Parkinsons disease, USP30, USP30 inhibitors

## Abstract

Mitochondrial dysfunction is implicated in Parkinson disease (PD). Mutations in Parkin, an E3 ubiquitin ligase, can cause juvenile-onset Parkinsonism, probably through impairment of mitophagy. Inhibition of the de-ubiquitinating enzyme USP30 may counter this effect to enhance mitophagy. Using different tools and cellular approaches, we wanted to independently confirm this claimed role for USP30. Pharmacological characterisation of additional tool compounds that selectively inhibit USP30 are reported. The consequence of USP30 inhibition by these compounds, siRNA knockdown and overexpression of dominant-negative USP30 on the mitophagy pathway in different disease-relevant cellular models was explored. Knockdown and inhibition of USP30 showed increased p-Ser65-ubiquitin levels and mitophagy in neuronal cell models. Furthermore, patient-derived fibroblasts carrying pathogenic mutations in Parkin showed reduced p-Ser65-ubiquitin levels compared with wild-type cells, levels that could be restored using either USP30 inhibitor or dominant-negative USP30 expression. Our data provide additional support for USP30 inhibition as a regulator of the mitophagy pathway.

## Introduction

Mitochondrial homeostasis is important for the survival of healthy cells. Indeed, mitochondrial dysfunction has been linked to several diseases including neurodegenerative disorders such as Parkinson's disease (PD) [1–5]. PD is a chronic, progressive neurodegenerative disease that has been linked mechanistically and genetically to alterations in mitochondrial homeostasis [1,6–8]. It is thought that damaged mitochondria accumulate in neuronal cells, leading to neurotoxicity. In PD the dopaminergic neurones in the substantia nigra display increased neurotoxicity accompanied by mitochondrial dysfunction, leading to the characteristic motor dysfunction [9]. Thus, it has been proposed that enhanced autophagic removal of damaged mitochondria by a process called mitophagy may be a therapeutic approach for the treatment of PD [5,10,11].

The hypothesis that mitophagy enhancement could be of benefit in PD was substantiated by the finding that mutations in key components of the mitophagy pathway such as *PINK1* or *PRKN* (Parkin) can cause familial autosomal recessive Parkinsonism [12,13]. Mitophagy is a multi-step process that ensures the removal of damaged mitochondria (e.g. those with loss of membrane potential) through the stabilisation of the PTEN-induced kinase 1 (PINK1) in the outer mitochondrial membrane to result in phosphorylation of PINK1 targets, most notably ubiquitin and the ubiquitin-like domain (Ubl) in Parkin at homologous serine 65 residues [14]. Phospho-ubiquitin (p-Ser65-Ub) recruits and activates the Parkin E3 ubiquitin ligase, resulting in ubiquitination of target proteins such as translocase of outer mitochondrial membrane 20 (TOM20) and mitofusin-2 (Mfn-2) at the mitochondria outer membrane [15]. Ubiquitinated mitochondria in turn recruit mitophagy receptors such as optineurin (OPTN) and nuclear dot protein 52 (NDP52) to the site of mitochondrial damage [16], engaging the autophagy machinery to initiate the formation of autophagosomes. Subsequently, the autophagosome engulfs damaged mitochondria and delivers them to the lysosome for degradation.

PINK1/Parkin-mediated mitophagy has been proposed as a pathway containing modulatable targets for drug discovery strategies aimed at enhancing mitophagy in various diseases, including ageing and PD [17–20]. In addition to PINK1 and Parkin, de-ubiquitinating (DUB) enzymes that regulate the stability of key mitophagy proteins have also been suggested as possible drug targets [21,22]. Several DUB's have been shown to influence mitochondrial homeostasis, including ubiquitin-specific peptidase 8 (USP8) [23], USP15 [24], USP30 [25], USP33 [26] and USP35 [27].

Among these, USP30 has been investigated in depth and knockdown of USP30 was demonstrated to overcome defects in Parkin activity and enhance survival of dopaminergic neurones [25]. USP30 is localised in the mitochondrial outer membrane and was originally believed to act as the DUB that antagonises the PINK1/Parkin pathway [25,28]. However, recent findings have suggested that USP30 acts at earlier stages, possibly as a gatekeeper for mitochondrial ubiquitination, acting to dampen Parkin activity and also to prevent the unscheduled initiation of mitophagy and keep the system under tight control [29]. It has also been suggested that a key role for USP30 is to facilitate and quality control-check protein import in mitochondria [30,31]. Recently, it has been shown that USP30 can also regulate peroxisome function [29].

The USP30 reports so far have used certain pharmacological tools, different genetic approaches and various cell lines. We wanted to conduct independent studies, searching for complementary pharmacological agents and approaches to test effects of USP30 perturbation on mitophagy in additional and different cell types. USP30 effects on mitophagy may also be dependent on the various methods to induce mitophagy. Here, several pharmacological USP30 inhibitors were characterised for their potency and selectivity against a panel of DUBs. The most selective compound was further characterised for its ability to modulate PINK1/Parkin-mediated mitophagy. Tandem Ubiquitin Binding Entities (TUBEs), p-Ser65-Ub and mitoKeima were assessed in different cell models including iPSC-derived neurones and patient-derived fibroblast with pathogenic Parkin mutations. We report the results of our investigations below.

## Methods

### Materials and compound synthesis

All chemicals and compounds were purchased from Sigma–Aldrich unless otherwise specified. USP30 inhibitors were derived from patent ID: WO 2016/156816 and WO 2017/103614 and synthesised in-house.

### Antibodies

The following antibodies were used for Western blotting: anti-Mfn2 (Abcam, ab66889 and ab124773, 1 in 500), anti-total ubiquitin (FK2, Enzo, BML-PW8810-0100, 1 in 500), anti-phospho-Ser65-ubiquitin (Millipore, ABS1513-I, 1 in 300), anti-GAPDH (CST, 2118L, 1 in 2000), anti-OXPHOS cocktail (Abcam, ab110411, 1 in 500), anti-VDAC1 (Abcam, ab154856, 1 in 1000), anti-USP30 (Sigma, HPA016952, 1 in 500), anti-USP30 (Santa Cruz, sc-515235, 1 in 100), anti-Vinculin (Abcam, ab129002, 1 in 10 000), anti-GFP (ab13970, 1 in 1000)).

The following antibodies were used for immunocytochemistry (ICC): anti-phospho-Ser65-ubiquitin (Millipore, ABS1513-I, 1 in 1000), anti-phospho-Ser65-ubiquitin (CST, 62802, 1 in 1000), anti-HSP60 (Abcam, ab128567, 1 in 1000), anti-tyrosine hydroxylase (Millipore, AB1542, 1 in 100), anti-Beta III tubulin (Biolegend, 801201, 1 in 2000), anti-GFAP (CST, 3670S, 1 in 200).

### Cell culture

All cell culture media and supplements were purchased from Fisher Scientific unless otherwise specified. SHSY5Y (human neuroblastoma) cells were purchased from ATCC and cultured in Dulbecco's Modified Eagle Medium (DMEM; high glucose plus GlutaMAX), supplemented with 10% fetal bovine serum (FBS) and 100 U/ml penicillin/100 µg/ml streptomycin (Gibco). Parkin mutant fibroblasts were obtained from the NINDS Repository. Parkin^+/+^ control (ND36320), Parkin^+/R275W^ (ND29369) and Parkin^R275W/R275Q^ (ND40072) were grown in DMEM (high glucose, plus GlutaMAX), containing 10% fetal calf serum (FCS; Labtech), 1 mM sodium pyruvate and 100 U/ml penicillin/100 µg/ml streptomycin.

iDOPA neurones and iAstrocytes (both Cellular Dynamics International) were plated on a matrix of poly-l-ornithine (Sigma, 0.01%) and laminin (Sigma, 10 µg/ml in D-PBS) in clear bottom 96 well CellCarrier Ultra plates (PerkinElmer) at a density of 100 000 and 10 000 cells per well, respectively. Cells were left to mature over 5 days, feeding every 2–3 days before compound treatment.

### USP30 activity assay

USP30 inhibitors were dispensed into black, clear bottom, low binding 384 well plates (Greiner) using the ECHO 550 (Labcyte) liquid handler. An amount of 75 nl of compound was dispensed in 100% DMSO. Total reaction volume was 30 µl producing an USP30 inhibitor top concentration of 25 µM. His-tagged, 2× concentrated recombinant human USP30 protein (rhUSP30; amino acids 57–517 of the full-length protein, and a C-terminal 6-His tag, Sf 21 (baculovirus)-derived human USP30 protein) (10 nM; final assay concentration = 5 nM; Boston Biochem) was prepared in USP30 activity assay buffer (50 mM Tris base pH 7.5, 100 mM NaCl, 0.1 mg/ml BSA (Sigma, A7030), 0.05% Tween 20, 1 mM DTT) and 15 µl dispensed into compound containing assay plate using the Multidrop dispenser (Thermo Scientific) and incubated for 30 min at room temperature. Following incubation, 15 µl of 2× concentrated ubiquitin-rhodamine 110 (Ub-Rho110) (200 nM, final assay concentration = 100 nM; Boston Biochem), prepared in USP30 activity assay buffer, was dispensed into the compound-rhUSP30 containing plate and fluorescence immediately read on the FLIPR TETRA plate reader (Molecular Devices). Fluorescence was recorded every 30 s over 1 h and intensity was analysed.

### Di-ubiquitin cleavage assay

His-tagged rhUSP30 was diluted to 600 nM in USP30 activity assay buffer (see above) and incubated with USP30Inh-1 where appropriate for 15 min at room temperature. Lysine-6-di-ubiquitin (K6-di-Ub) was solubilised to 30 µM following manufacturer's instructions and further diluted to 10 µM in USP30 activity assay buffer. His-tagged rhUSP30-USP30Inh-1 was combined with a K6-di-Ub at a ratio 3 : 1, yielding final assay concentration of 450 nM and 2.5 µM, respectively. The plate was centrifuged, covered and incubated for 2 h at 37°C. The reaction was stopped following the addition of 4× sample buffer (containing 10% beta mercaptoethanol (BME; Thermo Fisher)) to each well and heated to 95°C for 10 min. Proteins were resolved by SDS–PAGE, the gel was fixed (50% methanol, 7% acetic acid in water) for 15 min and washed three times in water. GelCode Blue (Fisher Scientific) was used to stain proteins for 1.5 h to overnight, de-stained using water and imaged using the Li-Cor Odyssey CLx. Mono- and di-ubiquitin band intensity was quantified using Image Studio (Licor).

### DUB selectivity profiling

Selectivity profiling of 41 DUB enzymes was performed at Ubiquigent (Dundee, U.K.) using the DUBprofiler™ platform and Ub-Rho110-glycine substrate based-assay.

### SDS–PAGE and Western blot

Cells were washed in cold D-PBS prior to lysis on ice using lysis buffer (1% NP40, 100 mM Tris pH8, 100 mM NaCl, 10% glycerol, 5 mM EDTA, cOmplete^TM^ EDTA-free Protease Inhibitor Mixture, Roche Applied Science) supplemented with 20 mM N-ethylmaleimide. Lysates were cleared by centrifugation at 15 000×***g*** at 4°C, and the resulting pellet was discarded. Total protein concentrations were determined using Pierce BCA protein assay kit (Thermo Fisher Scientific), and lysates were diluted to approximately equal concentrations before addition of 4× sample buffer (containing 10% BME) with immediate boiling at 95°C for 10 min. Proteins were separated by 4–12% bis-tris SDS–PAGE (Invitrogen) in 1× MOPS or MES SDS running buffer (Invitrogen) at 100 V, and transferred (1× Transfer Buffer (Invitrogen), 20% methanol) onto Immobilon-FL PVDF membrane (Millipore) for 1.5 h at 100 V. Membranes were blocked in Odyssey blocking buffer TBS (Licor) or 5% non-fat milk or 1% BSA (Sigma, A7906) for 1 h before overnight incubation at 4°C with primary antibodies. Membranes were scanned the following day after 1 h incubation with secondary antibodies using the Odyssey CLx Imaging System or ImageQuant system (Bio-Rad). The following secondary antibodies were used: HRP anti-rabbit (CST, 7074S) and anti-mouse (CST, 7076S) or LI-COR IRDye® 800CW (anti-rabbit) and IRDye 680RD (anti-mouse).

### Target engagement using activity-based probe; Biotin-Ahx-Ub-PA

SHSY5Y cells were plated at 0.5 × 10^6^ cells per well in six well plates. Following compound incubation, cells were washed three times in ice-cold D-PBS (without Ca^2+^ and Mg^2+^) and lysed in HR buffer (50 mM Tris base, 250 mM sucrose, 5 mM MgCl_2_, 0.1% NP40, 0.5% CHAPS) with DTT (1 mM). 1x HALT™ Protease and Phosphatase Inhibitor Cocktail (Fisher Scientific) were added immediately before lysis. Cell lysates were sonicated three times using a probe tip sonicator (5 s; amplitude = 6, Soniprep 150 Plus, MSE) and centrifuged at 4°C for 10 min at 18 000×***g****.* Supernatants were collected and protein quantified by BCA assay and diluted to 1 mg/ml in HR lysis buffer.

Biotin-Ahx-Ub-PA (UbiqBio) was solubilised to 100 µM following manufacturer's instructions. An equivalent of 2.5 µM activity-based probe (ABP) per 100 µg protein lysate was incubated at room temperature for 1 h with constant agitation. The assay was terminated following addition of 4× concentrated sample buffer (Licor), containing 10% BME. Samples were heated to 95°C for 10 min before separation by SDS–PAGE and Western blotting.

### Ubiquitinated protein enrichment using TUBEs

Fibroblasts were plated at 1 × 10^6^ cells per 10 cm dish and left to adhere overnight. The following day, USP30Inh-1 was added where appropriate and replenished with a full media change at day 3. At day 6, mitophagy was induced using FCCP (10 µM) for 2 h. Plates were washed twice in ice-cold D-PBS (without Ca^2+^ and Mg^2+^) and lysed in 300 µl NP40 lysis buffer (1% NP40, 100 mM Tris base pH 8, 100 mM NaCl, 10% glycerol, 5 mM EDTA, final concertation pH 8). 1× HALT™ Protease and Phosphatase Inhibitor Cocktail, 50 µM PR-619 and 10 mM N-ethylmaleimide (Fisher Scientific) were added immediately before lysis. Cells were scraped and homogenates collected.

Lysates were centrifuged at 4°C for 10 min at 18 000×***g*** and supernatant collected and input sample removed. An amount of 50 µl of Magnetic TUBE1 (LifeSensors) was added to 300 µl of cell lysate and the suspension left to rotate at 25 rpm overnight at 4°C. The following day, samples were placed on a magnetic rack and flow through sample removed. Remaining lysate was removed, and magnetic beads washed four times with ice-cold Tris buffered saline-0.1% Tween 20 (TBS-T). Following the final wash, protein was eluted using 40 µl of 1× Licor sample buffer (containing 2.5% BME) and heated for 10 min at 95°C. Proteins were resolved by SDS–PAGE and analysed by Western blotting.

### Immunocytochemistry

Cells were washed with D-PBS and ice-cold fixing solution (1 : 1; acetone: methanol) was added, and plates incubated for 2 min on ice. Cells were washed twice using D-PBS. Cells were blocked for 1 h at room temperature (3% BSA (Sigma, A3803) in D-PBS). Primary antibodies were diluted in blocking buffer, added to cells and plates were incubated at 4°C overnight. Cells were washed twice in TBS-T and secondary antibody solution was added: anti-mouse Alexa-488 and anti-rabbit Alexa-647 (Fisher Scientific) diluted 1 in 1000; nuclear stain (Hoechst 33342, final concentration of 2 µg/ml) diluted in blocking buffer and incubated for 1 h at room temperature. Cells were washed twice in TBS-T and imaged using the PerkinElmer Opera Phenix.

### Measurement of mitophagy levels by using the mitoKeima reporter

For the Keima reporter assay, SHSY5Y cells were transfected with the mitochondria-targeted monomeric Keima-Red (mitoKeima) (Medical and Biological laboratories Co., Ltd. AM-V0251) [32,33]. Stable cell lines were generated by culturing cells in media containing 500 µg/ml G418 (Sigma). For treatments with USP30Inh-1, cells were seeded into 384 well CellCarrier Ultra plates (PerkinElmer). The following day cells were treated with different concentrations of USP30Inh-1 for 3 days. On the assay day, media was replenished with DMEM lacking phenol red (Thermo Scientific, containing 10% FBS, 100 U/ml penicillin/100 µg/ml streptomycin and 1× L-glutamine) which contained fresh compound and then mitophagy was induced by adding (final concentrations) 10 µM carbonyl cyanide m-chlorophenyl hydrazone (CCCP) or 1/1 µM antimycin A/oligomycin (A/O). 2 µg/ml Hoechst 33342 (Thermo Fisher) was included in order to identify nuclei. Images were acquired on the PerkinElmer Opera Phenix high content confocal microscope using the 63x water objective with temperature and CO_2_ controls enabled. Excitation wavelengths and emission filters used were as follow: Cytoplasmic Green Keima: 488 nm, 650–760 nm; Lysosomal red Keima: 561 nm, 570–630 nm; Hoechst: 375 nm, 435–480 nm. Images were analysed by using a CellProfiler pipeline as follows: Cells, cytoplasmic green and lysosomal red mitoKeima areas were segmented and the area occupied by the two respective mitoKeima signals was determined. The mitophagy index was calculated as the ratio of the total lysosomal red mitochondria area divided by the total cytoplasmic green mitochondria area.

### Transfection

For mRNA transfection in fibroblasts, mRNAs were synthesised *de novo* (Trilink Biotechnologies) using cDNA sequences for *USP30* (accession NM_032663) and introducing point mutation c.G230C p.C77S, and *PRKN* (accession NM_004562.3). An amount of 7 µg mRNA was transfected into 0.5 × 10^6^ fibroblasts using Cell Line Nucleofector Kit V (Lonza) and AMAXA program X-001 following the manufacturer's instructions. Cells were assayed 18–20 h later and EGFP expression was confirmed by imaging.

USP30 knockdown in SHSY5Y cells was conducted as previously described [29] by following the ‘double hit’ approach where cells are transfected twice over a period of 7 days. Briefly, cells were seeded in either 12-well plates (0.1 × 10^6^ cells) or 96-well CellCarrier Ultra plates (8000 cells/well; PerkinElmer) or Seahorse 96 well plate (15 000 cells/well; Agilent) before transfecting with 40 nM ONTARGETplus Non-targeting oligo siRNA (NT1 siRNA: 5′-UGGUUUACAUGTCGACUAA-3′, Dharmacon) or with a pool of two different USP30 siRNAs (D1: 5′-CAAAUUACCTGCCGCACAA-3′; D3, 5′-ACAGGAUGCUCACGAAUUA-3′, Dharmacon; siUSP30) by using Lipofectamine RNAiMAX (Thermo Scientific) following the manufacturer's instructions.

### Oxygen consumption rate measurement

Mitochondrial function in SHSY5Y cells was measured by using the Seahorse XFe96 Extracellular Flux Analyser (Agilent). For treatment with USP30Inh-1 8000 cells per well were seeded in the Seahorse 96 well plate and left to attach overnight. Next day, cells were treated with different USP30Inh-1 concentrations for 24 h. On the assay day, cell culture media was washed and replaced with fresh assay media (XF basic media; Agilent), supplemented with 10 mM glucose, 2 mM l-glutamine and 1 mM sodium pyruvate (pH was adjusted to 7.4). The plate was incubated at 37°C for equilibration for 1 h before loading to the analyser. Mitochondrial respiration was measured by using the Mito-Stress Test (Agilent) as per manufacturer's instructions. Oligomycin (1 µM), FCCP (1.2 µM), rotenone (1 µM) and antimycin A (1 µM) were sequentially added to cells to determine mitochondrial respiration parameters. For the normalisation step, 1 µg/ml Hoechst 33342 (Thermo Fisher) was added to the cells and incubated for 10 min before imaging on the PerkinElmer Opera Phenix.

### Mitochondrial membrane potential

Mitochondrial membrane potential was measured using TMRM in re-distribution mode. Media was replaced with compound containing TMRM staining solution (50 nM tetramethyl rhodamine methyl ester (TMRM), 200 nM Mitotracker Green FM (MTG; Fisher Scientific), 2 µg/ml Hoechst) in complete cell culture media and left for 1 h at 37°C to equilibrate. Images were acquired using the PerkinElmer Opera Phenix with temperature and CO_2_ controls enabled.

### Data analysis and statistics

Data are presented as mean ± standard deviation (SD). Normalisation of the data allowed for control of inter-assay variability. Curve fitting was performed using GraphPad Prism version 6.05 for Windows. Statistical tests are indicated in figure legends. Statistical significance was assessed as being *P* < 0.05.

## Results

### Knockdown of USP30 enhances mitoKeima signal in SHSY5Y cells

**Figure 1.**
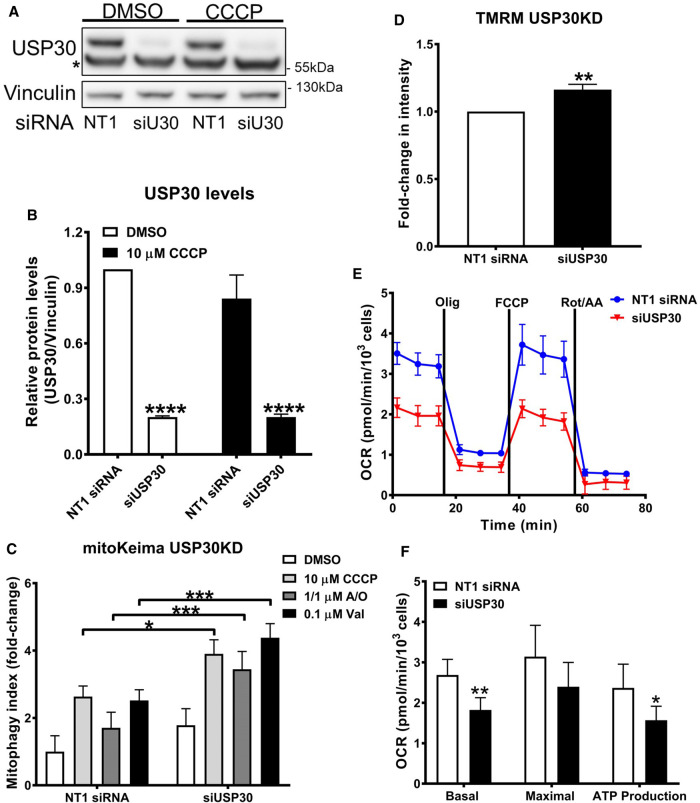
siRNA-mediated knockdown of USP30 enhances mitoKeima signal in SHSY5Y cells. (**A**) Representative immunoblotting of USP30 expression levels after 7 days transfection with siUSP30 (siU30) and a non-targeted (NT1) siRNA in SHSY5Y mitoKeima cells. Vinculin was blotted as a loading control. In parallel, incubation with 10 µM CCCP for 4 h was tested in order to determine if it affects USP30 protein expression in the SHSY5Y mitoKeima cells. * indicates a non-specific band coming from the used USP30 antibody. (**B**) Quantification of USP30 protein expression levels from three independent immunoblotting experiments as presented in (**A**). (**C**) USP30 was knocked down in SHSY5Y mitoKeima cells for 7 days before inducing mitophagy with 10 µM CCCP or 1 µM A/O or 0.1 µM Val for 7 h. Cells were imaged with the Opera Phenix. With all treatments, enhanced mitophagy was observed under the USP30 knockdown conditions. The mitophagy index was calculated as the fold change of the ratio of the total lysosomal red mitochondria area divided by the total cytoplasmic green mitochondria area from three independent experiments. (**D**) USP30 was knocked down in SHSY5Y cells for 7 days before incubation with 50 nM TMRM and imaging with the Opera Phenix. Fold change in TMRM intensity from three independent experiments was quantified. (**E**) Representative OCR trace as measured by the Seahorse^TM^ analyser in SHSY5Y cells, following USP30 knockdown for 7 days with siUSP30. (**F**) Basal respiration, maximal respiration and ATP production were determined based on the OCR measurement from 4 independent experiments. Data are pooled from 3–4 independent experiments. Error bars show means ± SD. * *P* < 0.05, ** *P* < 0.01, *** *P* < 0.001, **** *P*<0.0001. Data were analysed with Two-way ANOVA with Sidak's test for for C and F, and unpaired *t*-test for D.

It has been proposed that inhibition of USP30 could be a potential mechanism enabling discovery of therapeutic agents for PD [21,25,29]. Here, we explored USP30 inhibition via siRNAs and its effect when mitophagy was induced via different mitochondrial toxins. We used a pool of two different USP30 siRNAs (siUSP30) to achieve a greater than 80% decrease in the protein levels of USP30 in SHSY5Y cells (Figure 1A,B). The expression of USP30 protein was not affected by the addition of CCCP (Figure 1A,B). Next, we used SHSY5Y cells that stably express the mitoKeima reporter to investigate the effect of USP30 knockdown on mitophagy. After 7 days of USP30 knockdown, cells were treated with mitophagy inducers CCCP, A/O and valinomycin (Val). In cells treated with a non-targeted (NT1) siRNA, incubation with CCCP, A/O and Val for 7 h resulted in 2.6-fold, 1.7-fold and 2.7-fold increase in the mitoKeima signal (Figure 1C and Supplementary Figure S1). In the USP30 knockdown cells treated with the same mitochondrial toxins a further 2-fold statistically significant increase was observed (Figure 1C and Supplementary Figure S1). USP30 knockdown, in our hands, does not seem to significantly increase basal levels of mitoKeima signal as reported previously [29].

The effects of USP30 knockdown in SHSY5Y cells on mitochondrial function were assessed by measuring the mitochondrial inner membrane potential using TMRM and analysing mitochondrial respiration parameters. Knockdown of USP30 induced a small (16%) but statistically significant increase in TMRM (Figure 1D) and a decrease in the oxygen consumption rate (OCR; Figure 1E). Knockdown of USP30 significantly decreased basal respiration as well as oxygen consumed during oxidative phosphorylation (+Oligomycin; Figure 1F). Reduced respiration is in agreement with a recent study in primary hepatocytes from USP30 knockout mice [34]. In conclusion, we show that genetic knockdown of USP30 can increase mitophagy as indicated by the increased mitoKeima signal but may also affect distinct aspects of mitochondrial function in the SHSY5Y cells.

### Identification of potent and selective USP30 inhibitors

**Figure 2.**
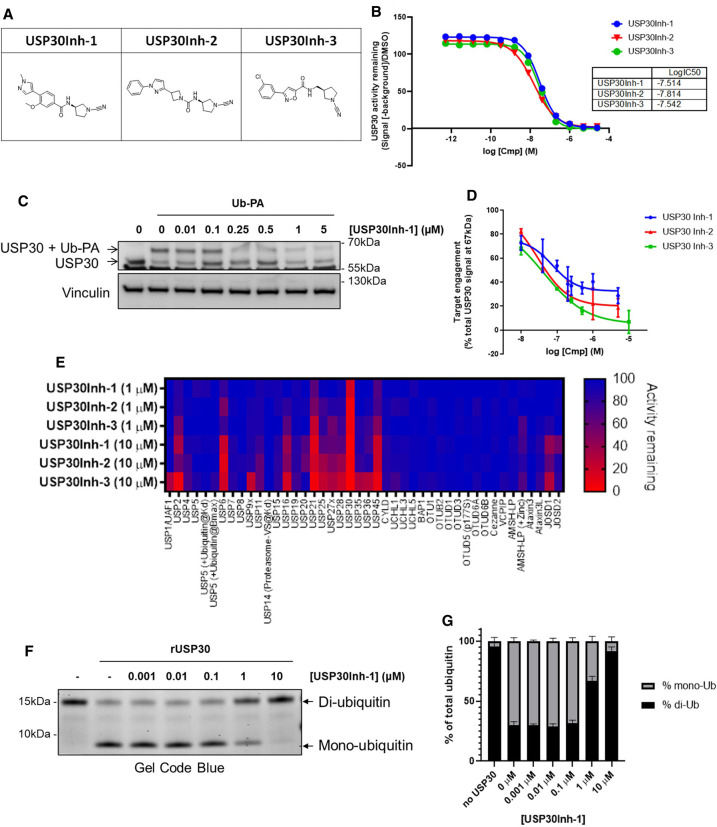
Identification of potent and selective USP30 Inhibitors. (**A**) Chemical structure of USP30Inh-1, 2 and 3. (**B**) Inhibition of the activity of recombinant human USP30 (rhUSP30) protein was tested by using 100 nM Ub-Rho110 as substrate when incubated with the indicated concentrations of USP30Inh-1, 2 and 3. (**C**) The Biotin-Ahx-Ub-PA was used with the ABP assay in order to determine in-cell target engagement of USP30 inhibitors. Representative immunoblotting for the USP30 expression when SHSY5Y cells were treated with the indicated USP30Inh-1 concentrations for 24 h before incubating cell lysates with 2.5 µM Ub-PA for 1 h at room temperature. Engagement of the probe is indicated by an ∼8 kDa shift in the molecular weight of USP30. (**D**) Quantification of the USP30 target engagement for USP30Inh-1, 2 and 3 from 2–4 independent experiments (USP30Inh-1, *n* = 4; USP30Inh-2, *n* = 3 and USP30Inh-6, *n* = 2). (**E**) DUB selectivity assay (DUBprofiler^TM^) was conducted using 1 and 10 µM of USP30Inh-1, 2 and 3. (**F**) The effect on the mono-Ub formation was assessed via SDS–PAGE where the indicated USP30Inh-1 concentrations were incubated for 2 h at 37°C with 450 nM rhUSP30 and 2.5 µM K6-di-Ub. (**G**) Changes in the di- and mono-Ub formation were quantified from three independent experiments.

Next, we aimed to recapitulate the effects we observed with knockdown of USP30 by using a pharmacological approach. To accomplish that, three structurally related small molecule USP30 inhibitors (which we term USP30Inh-1, -2 and -3) were synthesised based on compound structures published in patents: WO 2016/156816 and WO 2017/103614 (Figure 2A). Here, the cyano-amide functional group in the compounds is anticipated to form a covalent linkage with the catalytic cysteine within the USP30 active site, thereby inactivating the enzyme.

USP30 inhibitory activity was assessed biochemically using the fluorogenic artificial DUB substrate, ubiquitin-rhodamine 110 (Ub-Rho110) and recombinant USP30. USP30Inh-1, -2 and -3 all potently inhibited USP30-mediated cleavage of Ub-Rho110, with calculated IC_50_ values of between 15–30 nM (Figure 2B). To determine in-cell target engagement of USP30 inhibitors, the activity-based ubiquitin probe (ABP), Biotin-Ahx-Ub-propargylamide (PA) was used. The C-terminal PA electrophile forms a covalent linkage with the active site cysteine residue of DUB enzymes. Binding of Biotin-Ahx-Ub-PA to the USP30 is observed as a band shift with an ∼8 kDa increase in USP30 molecular weight following SDS–PAGE and immunoblot analysis (Figure 2C). USP30Inh-1, -2 and -3 were observed to reduce ABP engagement in a concentration-dependent manner, suggesting compounds compete with the ABP for access to the USP30 active site catalytic cysteine, are therefore cell permeable and capable of binding endogenous USP30 (Figure 2C,D). The selectivity of USP30 inhibitors was assessed using the Ubiquigent DUBprofiler™ service. USP30Inh-1, -2 and -3 demonstrated good selectivity against >40 known DUB enzymes at 1 µM. Decreased selectivity was observed for each compound at 10 µM, with greatest off-target inhibition against USP6, USP21 and USP45 for all three compounds (Figure 2E). USP30Inh-1 demonstrated the greatest selectivity based on this panel and was taken forward for further investigation.

*In vitro*, USP30 demonstrates preference for Lys6-linked ubiquitin chains [35,36]. To confirm inhibition, we assessed USP30-mediated cleavage of Lys6-linked di-ubiquitin chains upon incubation of the recombinant USP30 with the native substrate (K6-di-Ub). In the absence of inhibitor, USP30 efficiently cleaved K6-di-Ub, to yield mono-ubiquitin (Figure 2F, lane 2). USP30Inh-1 dose-dependently inhibited USP30-catalysed cleavage of K6-di-Ub (Figure 2F,G). Together, these data confirm USP30Inh-1 as a potent and, based on the studied DUBprofiler™ panel, moderately selective, cell-permeable small molecule inhibitor of USP30. In addition to our study, Phu *et al*. [31] and Rusilowicz-Jones *et al*. [37] have also shown that compounds containing the cyano-amide functional group have some off-target activity when using higher concentrations. These studies emphasise the need to carefully profile tool compounds used in cellular studies in order to avoid effects that could be attributable to modulation of other targets.

### Pharmacological inhibition of USP30 enhances mitoKeima signal in SHSY5Y cells

**Figure 3.**
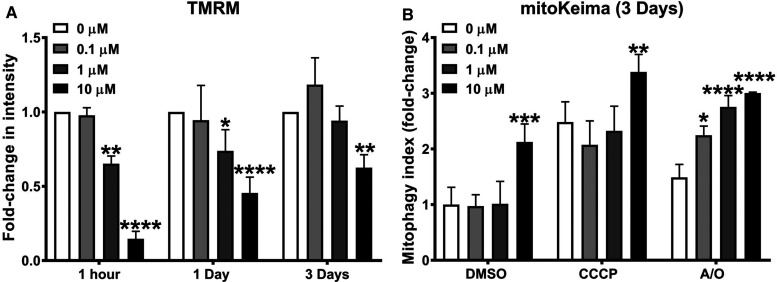
Pharmacological inhibition of USP30 enhances mitoKeima signal in SHSY5Y cells. (**A**) SHSY5Y cells were treated with the indicated USP30Inh-1 concentration for 1 h, 1 day and 3 days. On the assay day, fresh media containing the tested concentration as well as 50 nM TMRM was added and live images were acquired by the Opera Phenix. Fold change in TMRM intensity from three independent experiments was quantified. (**B**) SHSY5Y mitoKeima cells were treated with the indicated USP30Inh-1 concentration for 3 days. On the assay day, media was replaced with fresh medium containing fresh compound before inducing mitophagy with 1 µM A/O or 10 µM CCCP and live images were acquired with the Opera Phenix for a further 10 h. The mitophagy index was calculated as the fold change of the ratio of the total lysosomal red mitochondria area divided by the total cytoplasmic green mitochondria area from three independent experiments. Data are pooled from three independent experiments. Error bars show means ± SD. * *P* < 0.05, ** *P* < 0.01, *** *P* < 0.00, **** *P* < 0.0001. Data were analysed with Two-way ANOVA with Sidak's test.

In the SHSY5Y cells, the time- and concentration-dependent effect of USP30Inh-1 on mitochondrial inner membrane potential was measured using TMRM. Acute incubation (1 h) with 10 µM USP30Inh-1 caused a depolarisation of the mitochondrial membrane potential as more than 85% TMRM signal loss was observed. Increasing the incubation time to 1 and 3 days caused 40–50% depolarisation at 10 µM, suggesting that the compound displays mitochondrial toxicity at this concentration (Figure 3A). Treatment with 1 µM USP30Inh-1 also decreased TMRM after 1 h and 1 day incubation (45% and 25% decrease, respectively). No significant effect on the mitochondrial inner membrane potential was observed after 3 days treatment with 1 µM USP30Inh-1 (Figure 3A). Incubation with 0.1 µM USP30Inh-1 had no effect in the TMRM under all tested incubation times (Figure 3A). Therefore, we determined changes in mitophagy by measuring the mitoKeima signal in cells treated with USP30Inh-1 at the time point of 3 days. Prolonged incubation might be required in order to see changes in mitophagy after inhibition of USP30 which could be due to its suggested role in controlling the threshold of mitophagy [29,37].

Next, SHSY5Y mitoKeima cells were treated with 0.1, 1 and 10 µM USP30Inh-1 for 3 days (Figure 3B and Supplementary Figure S2). On the assay day, compound was added to the cells before inducing mitophagy by incubating cells with 1/1 µM A/O or 10 µM CCCP for 10 h. Incubation with 10 µM USP30Inh-1 demonstrated 2-fold significant increase in basal mitoKeima signal (+DMSO) and a further 1 to 1.4 -fold increase when CCCP or A/O was present (compared with 10 µM + DMSO; Figure 3B). The observed changes with 10 µM USP30Inh-1 could have resulted from the mitochondrial depolarisation observed using 10 µM treatment at that time point. On the other hand, cells treated with 0.1 and 1 µM USP30Inh-1, were able to significantly increase mitoKeima signal by 2.3 and 2.7-fold, respectively, but only when treated with A/O (Figure 3B). In conclusion, we have shown that, by carefully selecting a non-toxic concentration (0.1 µM), time of incubation conditions as well as the mitochondrial toxin, we can induce mitophagy by using a potent and selective USP30 inhibitor in SHSY5Y cells.

### USP30 inhibition increases p-Ser65-Ub in dopaminergic neurones and astrocytes

To establish whether USP30Inh-1 perturbs PINK1/Parkin-mediated mitophagy in a PD-relevant cell type, the abundance of p-Ser65-Ub in iPSC-derived midbrain dopaminergic (DA) neurone/astrocyte co-cultures was assessed. p-Ser65-Ub levels are a consequence of both the activity of PINK1 and Parkin, given the specificity of PINK1-dependent ubiquitin phosphorylation and the feed-forward mechanism of Parkin recruitment and poly-ubiquitin chain elongation [14].

**Figure 4.**
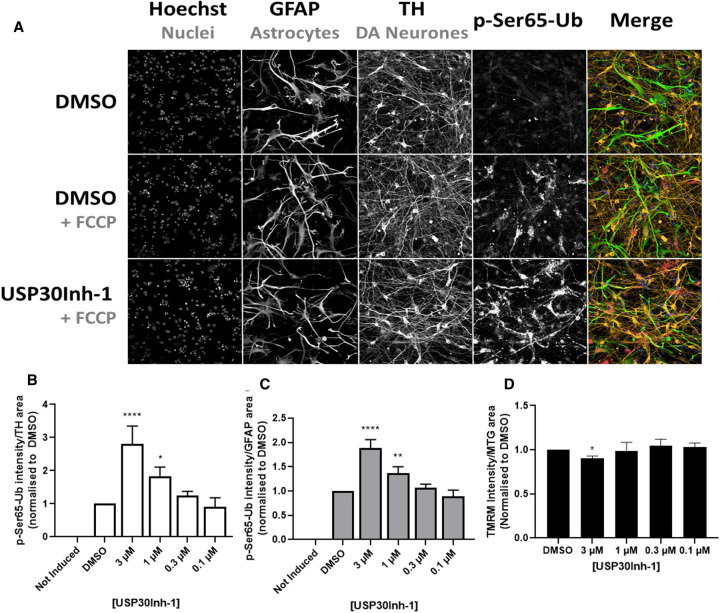
USP30 inhibition increases p-Ser65-Ub in dopaminergic neurones and astrocytes. (**A**) Representative immunostaining of p-Ser65-Ub in a co-culture of dopaminergic neurones and astrocytes. Cells were co-cultured for 5 days before incubating with 3 µM USP30Inh-1 for 4 more days. Mitophagy was induced by adding 10 µM FCCP for 2 h. Cells were immunostained with TH, GFAP and p-Ser65-Ub. (**B**) Immunoreactivity levels of p-Ser65-Ub in the dopaminergic TH-positive neurones was quantified from three independent experiments. (**C**) Immunoreactivity levels of p-Ser65-Ub in the astrocytes-GFAP positive cells was quantified from three independent experiments. (**D**) The dopaminergic neurones/astrocytes co-cultures were treated with the indicated USP30Inh-1 concentrations for 4 days before 50 nM TMRM/200 nM Mitotracker green (MTG) were added and imaged with the Opera Phenix. Intensity of TMRM from three independent experiments was quantified. Data are pooled from three independent experiments. Error bars show means ± SD. * *P* < 0.05, ** *P* < 0.01, **** *P* < 0.0001. Data were analysed with One-Way ANOVA with Dunnett's test.

A large increase in p-Ser65-Ub in both tyrosine hydroxylase (TH)-positive DA neurones and glial fibrillary acidic protein (GFAP)-positive astrocytes was observed following electron transport chain (ETC) uncoupling using FCCP (Figure 4A). Following 4 days incubation with USP30Inh-1, a concentration-dependent increase in p-Ser65-Ub immunoreactivity was detected within TH-positive (Figure 4B) and GFAP-positive (Figure 4C) regions, reaching statistical significance at 1 µM. Using TMRM, a small but significant decrease in mitochondrial inner membrane potential was measured within the whole-cell population but only when USP30Inh-1 reached 3 µM (Figure 4D). These data suggest that pharmacological inhibition of USP30 augments PINK1/Parkin-dependent signalling to result in increased levels of p-Ser65-Ub in this cellular system following mitochondrial uncoupling.

### Fibroblasts carrying heterozygote mutations in Parkin have reduced p-Ser65-Ub levels

We next aimed to identify PD patient-derived cell lines that carry genetic defects in mitophagy signalling, whereby USP30 perturbation may have functional, disease-relevant significance. Loss-of-function mutations in *PRKN* have been shown to cause autosomal recessive, juvenile-onset PD [12]. Patient-derived fibroblasts obtained from the NINDS Biorepository were genotyped against full-length, common variant *PRKN* (NG_008289.2). Fibroblasts were selected based on zygosity at arginine 275 (R275). The Parkin R275 mutation has been linked to Parkinson's disease through its severity of causing disruption in mitochondrial clearance [38].

Both heterozygote and compound heterozygote mutations (Parkin^+/R275W^ and Parkin^R275W/R275Q^) were confirmed using Sanger sequencing. Parkin mutations were not found within the control line (common variant; Parkin^+/+^).

**Figure 5.**
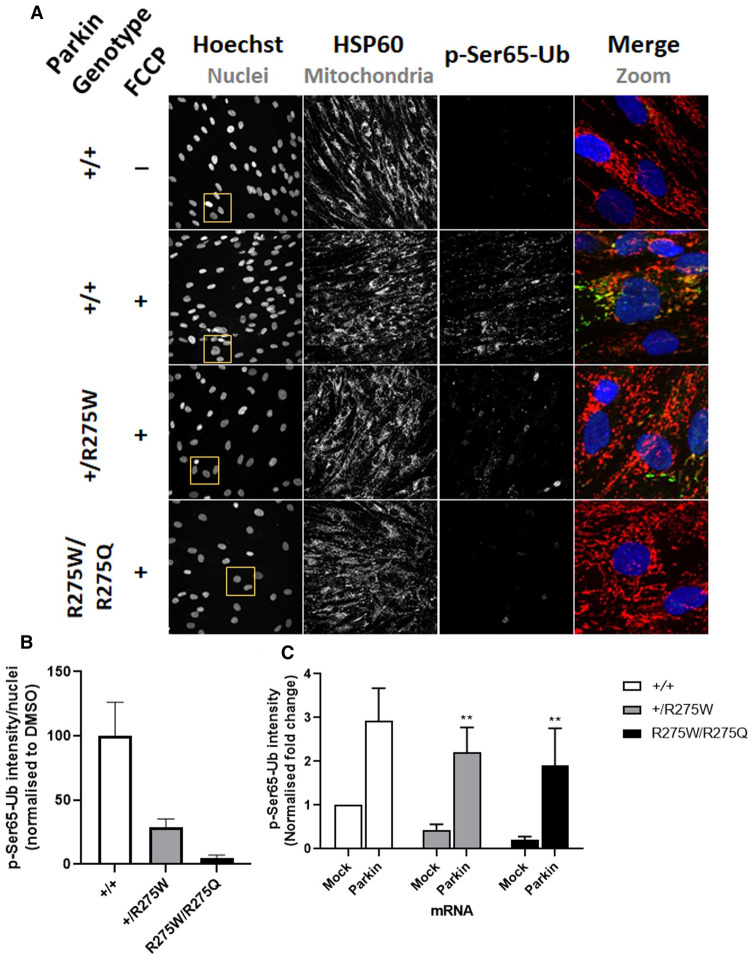
Parkin fibroblasts carrying heterozygote pathogenic mutations have reduced p-Ser65-Ub levels. (**A**) Representative images of the p-Ser65-Ub and HSP60 immunostaining in Parkin^+/+^, Parkin^+/R275W^ and Parkin^R275W/R275Q^ fibroblasts. Mitophagy was induced by adding 10 µM FCCP for 2 h. (**B**) Immunoreactivity of p-Ser65-Ub was quantified from three independent experiment in the fibroblasts from (**A**) where a genetic dose-response was observed. Expression levels were normalised to DMSO Parkin^+/+^ treated cells. (**C**) The Parkin^+/+^, Parkin^+/R275W^ and Parkin^R275W/R275Q^ fibroblasts were transfected with a Parkin WT mRNA and Luciferase (Luc) mRNA as a control for 20 h before inducing mitophagy for 2 h with 10 µM FCCP. Levels of p-Ser65-Ub were quantified from three experiments. The expression levels were normalised to mock transfected Parkin^+/+^ cells. Data are pooled from three independent experiments. Error bars show means ± SD. ** *P*<0.01. Data were analysed One-Way ANOVA with Dunnett's test.

To study the role of Parkin activity on Ser65-Ub phosphorylation in the patient-derived fibroblasts, p-Ser65-Ub immunoreactivity was measured and quantified (Figure 5A,B). A robust increase in p-Ser65-Ub was observed following mitochondrial ETC uncoupling by using FCCP in Parkin^+/+^ fibroblasts, suggesting an active PINK1/Parkin pathway (Figure 5A). Reduced p-Ser65-Ub immunoreactivity was determined in the Parkin^+/R275W^ cells compared with Parkin^+/+^, with negligible levels measured in the Parkin^R275W/R275Q^ line (Figure 5A). A genotype-phenotype relationship, determined by allelic frequency of *PRKN* mutation was observed with respect to p-Ser65-Ub immunoreactivity in the patient-derived fibroblasts (Figure 5B).

We next aimed to rescue p-Ser65-Ub deficits by re-introducing functional Parkin. A full-length mRNA that will express Parkin was transfected into each fibroblast line with high efficiency. p-Ser65-Ub immunoreactivity was then assessed following mitochondrial uncoupling with FCCP (Supplementary Figure S3). Re-expression of Parkin, increased p-Ser65-Ub abundance in all genotypes to similar levels (Figure 5C), suggesting the p-Ser65-Ub deficits observed are likely Parkin-mediated and there are no dominant-negative effects of the mutations. Together, these data suggest Parkin mutant fibroblasts present a valuable model by which to study genetic defects in PINK1/Parkin-mediated mitophagy and to further understand disease relevance of USP30 inhibition.

### Over-expression of USP30-C77S enhances p-Ser65-Ub in Parkin heterozygote fibroblasts

**Figure 6.**
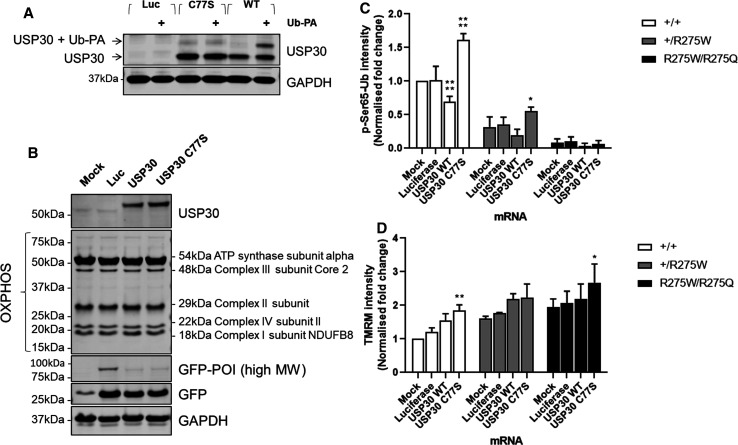
Over-expression of USP30-C77S enhances p-Ser65-Ub in Parkin heterozygote fibroblasts. (**A**) Lysates from the Parkin^+/+^ fibroblasts which have been transfected with USP30-WT mRNA and USP30-C77S mRNA for 20 h were incubated with 2.5 µM Ub-PA for 1 h at room temperature. Engagement of probe was assessed via immunoblotting for USP30 where engagement was observed only in the cells overexpressing USP30-WT and not USP30-C77S. (**B**) Lysates from the Parkin^+/+^ fibroblasts transfected with USP30-WT or USP30-C77S mRNA for 20 h were immunoblotted for both protein components of the mitochondrial respiratory complexes and, to determine transfection efficiency, GFP. No effect on the expression levels of the components of the mitochondrial respiratory complexes was observed under the tested conditions. The Parkin^+/+^, Parkin^+/R275W^ and Parkin^R275W/R275Q^ fibroblasts were transfected with the USP30-WT and USP30-C77S mRNA for 20 h before conducting immunostaining for p-Ser65-Ub. (**C**) The immunoreactivity of p-Ser65-Ub was quantified from 3–5 independent experiments. Expression levels were normalised to mock transfected Parkin^+/+^ cells. (**D**) Same conditions as (**C**) were tested but cells were incubated with 50 nM TMRM/200 nM MTG before imaging on the Opera Phenix. Intensity of TMRM from 3–5 independent experiments was quantified. Expression levels were normalised to mock transfected Parkin^+/+^ cells. Data are pooled from 3–5 independent experiments. Error bars show means ± SD. * *P* < 0.05, ** *P* < 0.01, **** *P* < 0.0001. Data were analysed One-Way ANOVA with Dunnett's test.

We next aimed to understand the effect of USP30 perturbation on PINK1/Parkin-mediated mitophagy in Parkin compromised cells. mRNA that express the wild type or catalytically dead (dominant-negative, C77S mutation) USP30 (USP30-Myc-P2A-eGFP) was transfected with high efficiency into patient-derived Parkin mutant fibroblasts. Expression and mitochondrial localisation of both epitope-tagged WT and C77S USP30 were confirmed by co-staining with the mitochondrial marker HSP60 (Supplementary Figure S4). To confirm the C77S mutation in USP30 caused loss-of-function, USP30 activity was assessed using the ABP, Biotin-Ahx-Ub-PA. USP30-C77S failed to bind the ABP as a band shift was not observed after SDS–PAGE and immunoblotting with antibody toward USP30. In contrast USP30-WT engaged the ABP, resulting in an ∼ 8 kDa band shift, suggesting the WT transcript generates active enzyme (Figure 6A). Neither WT nor catalytically dead USP30 had any effect on mitochondrial ETC subunit protein expression, and robust GFP expression at ∼27 kDa was observed following all mRNA transfections, proving efficient processing of the P2A sequence (Figure 6B).

We next assessed the effect of USP30 overexpression on p-Ser65-Ub immunoreactivity following mitochondrial uncoupling. Expression of USP30-WT significantly decreased p-Ser65-Ub abundance compared with firefly luciferase control in Parkin^+/+^ cells (Figure 6C and Supplementary Figure S5). The same trend was observed in the Parkin^+/R275W^ line; however, statistical significance of effects was not reached (Figure 6C). In contrast, expression of the USP30-C77S produced a significant increase in p-Ser65-Ub abundance in both the Parkin^+/+^ and Parkin^+/R275W^ lines (Figure 6C). No change in p-Ser65-Ub abundance was observed in the Parkin^R275W/R275Q^ (Figure 6C). TMRM analysis revealed a small apparent hyperpolarisation following USP30-C77S over-expression in Parkin^+/+^ and Parkin^R275W/R275Q^ lines (Figure 6D), potentially an imaging artefact due to observed morphological changes associated with the over-expression. Given inner membrane depolarisation is necessary to recruit and activate Parkin, we anticipate this will have little effect on p-Ser65-Ub abundance. Taken together, USP30, wild type or C77S mutant, can directionally impact p-Ser65-Ub levels, an event dependent on functional Parkin.

### Enhanced p-Ser65-Ub levels upon pharmacological inhibition of USP30 in Parkin heterozygote fibroblasts

**Figure 7.**
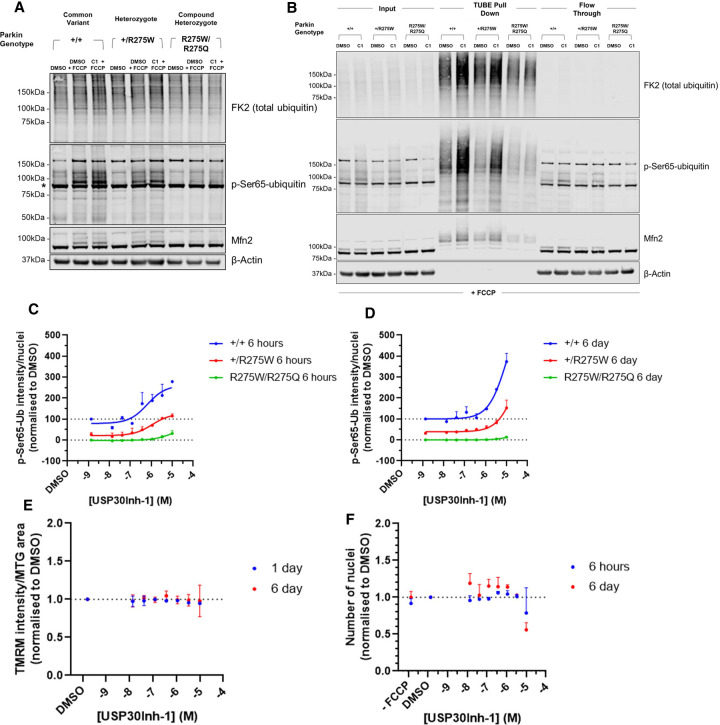
Enhanced p-Ser65-Ub levels upon pharmacological inhibition of USP30 in Parkin heterozygote fibroblasts. (**A**) The Parkin^+/+^, Parkin^+/R275W^ and Parkin^R275W/R275Q^ fibroblasts were treated with 3 µM USP30Inh-1 for 6 days before inducing mitophagy with 10 µM FCCP for 2 h. Lysates were immunoblotted for total ubiquitin, p-Ser65-Ub and Mfn2. *indicates a non-specific band coming from the used p-Ser65-Ub antibody. (**B**) Same conditions as at (**A**) but TUBE enrichment was used to enhance the levels of poly-ubiquitinated proteins before conducting immunoblotting. The Parkin^+/+^, Parkin^+/R275W^ and Parkin^R275W/R275Q^ fibroblasts were treated with the indicated concentrations of USP30Inh-1 for (**C**) 6 h or (**D**) 6 days before inducing mitophagy with 10 µM FCCP for 2 h. The immunoreactivity of p-Ser65-Ub was quantified from three independent experiments for each condition. The expression levels were normalised to DMSO Parkin^+/+^ treated cells (+/+ = 100% and Parkin^R275W/R275Q^ = 0%). (**E**) The Parkin^+/+^ fibroblasts were treated with the indicated concentrations of USP30Inh-1 for 1 day or 6 days. On the assay day, cells were incubated with 50 nM TMRM/200 nM MTG before imaging on the Opera Phenix. The intensity of TMRM from three independent experiments was quantified and no change was observed. (**F**) The Parkin^+/+^ fibroblasts were treated with the indicated concentrations of USP30Inh-1 for 6 h or 6 days. Cells were counted after staining for nuclei and average from three independent experiment was calculated. Reduced numbers of nuclei and therefore cells was observed only at 10 µM USP30Inh-1 after 6 days incubation.

We next determined the effects of pharmacological inhibition of USP30 in the Parkin mutant fibroblasts. Western blot analysis revealed increased p-Ser65-Ub following FCCP in both the Parkin^+/+^ and Parkin^+/R275W^ fibroblasts, and slightly enhanced following 6 days incubation with USP30Inh-1 (Figure 7A). Parkin^R275W/R275Q^ cells displayed minimal increases in p-Ser65-Ub following FCCP, with no effect of USP30Inh-1 (Figure 7A). We also looked into Mfn2 which is a well-characterised Parkin substrate [39,40]. Following induction of mitophagy, Mfn2 is rapidly extracted from the mitochondrial outer membrane and turned over by the proteasome [41]. In Parkin^+/+^ fibroblasts, Mfn2 protein levels decreased, concurrent with a robust increase in Mfn2 ubiquitination after addition of FCCP (Figure 7A, Lane 2). Reduced Mfn2 abundance and ubiquitination was evident in the Parkin^+/R275W^ cells compared with Parkin^+/+^ (Figure 7A, Lane 5), with negligible effects observed in the Parkin^R275W/R275Q^ line (Figure 7A, Lane 8). The Parkin genotype determined the degree of Mfn2 ubiquitination, with strong mono-ubiquitination in the Parkin^+/+^ fibroblasts and absence of any ubiquitinated species in the Parkin^R275W/R275Q^ line (Figure 7A). In all genotypes, under the conditions of the experiments, no effect of USP30Inh-1 was observed on Mfn2 mono-ubiquitination.

To further understand ubiquitination dynamics in response to USP30 inhibition, ubiquitin pull-down was performed using TUBEs. TUBEs enrich for poly-ubiquitinated proteins, with lower affinity for mono-ubiquitinated [42] and a clear increase in total poly-ubiquitin can be detected following TUBE pull down (Figure 7B). In the presence of FCCP, poly-ubiquitinated species of Mfn2 were observed with TUBE pull-down in both Parkin^+/+^ and Parkin^+/R275W^ lines, the greatest levels found in the Parkin^+/+^ cells (Figure 7B). In both Parkin^+/+^ and Parkin^+/R275W^ fibroblasts, the Parkin genotype-dependent increase in Mfn2 poly-ubiquitination was further enhanced with 6 days treatment of USP30Inh-1 (Figure 7B). In the Parkin^R275W/R275Q^ fibroblasts, Mfn2 poly-ubiquitination was not enhanced following incubation with USP30Inh-1 (Figure 7B). We identified FCCP/USP30Inh-1-dependent increases in Mfn2 and p-Ser65-Ub correlated with Parkin genotypes.

To confirm our observations and understand the kinetics associated with the ubiquitination response, Parkin mutant fibroblasts were incubated with USP30Inh-1 for either 6 h or 6 days and levels of p-Ser65-Ub were assessed by immunocytochemistry following FCCP treatment. As observed in Figure 5B, a Parkin genotype-dependent response was observed in p-Ser65-Ub immunoreactivity. At both 6 h and 6 days incubation, a USP30Inh-1 concentration-dependent increase in p-Ser65-Ub immunoreactivity was observed, correlating with severity of Parkin mutation (Figure 7C,D). In the Parkin^+/+^ fibroblasts, USP30Inh-1-dependent changes were greater following 6 days incubation and independent of any changes in mitochondrial inner membrane potential as measured using TMRM (Figure 7E). A small increase in p-Ser65-Ub was observed in the Parkin^R275W/R275Q^ fibroblasts, potentially suggesting some remaining functionality in Parkin. Notably, with 6 days treatment at 10 μM USP30Inh-1 but not at lower concentrations, a ∼40% decrease in cell number, was observed in the Parkin^+/+^ fibroblasts (Figure 7F), potentially reflecting increased cell death, cell detachment or decreased cell proliferation. Taken together, USP30 inhibition can, under appropriate conditions with careful selection of concentrations and time of incubation, rescue ubiquitination deficits in patient-derived Parkin^+/R275W^ fibroblasts to the levels of Parkin^+/+^. Functional Parkin is therefore required for USP30 inhibition to positively modulate the mitophagy cascade.

## Discussion

Mitochondrial dysfunction is a prominent pathological feature of both sporadic and familial PD [6,43,44]. PINK1 and Parkin are sensor and amplifier proteins integral to removing damaged mitochondrial by mitophagy [45–47]. The association between mutations in PINK1 and Parkin and the development of PD, suggests that defective mitophagy and accumulation of damaged mitochondria are key factors involved in the aetiology of disease. In addition to genetic deficits implicated in mitophagy, mitochondrial dysfunction and reduced rates of mitophagy are evident in sporadic PD [48–50], again linking mitochondrial health and clearance processes to PD pathophysiology. Oxidative stress and bioenergetic failure are recognised phenotypes of PD *in vivo* and *in vitro* [51,52] and improving the efficiency of mitochondrial quality control will likely prevent inappropriate generation of mitochondrially-derived oxidative intermediates and cellular damage [10]. Therefore, enhancement of mitochondrial clearance by mitophagy has been proposed as a disease-modifying strategy in PD. USP30 has been suggested as a potential target for enhancing mitophagy since it is the main DUB that is localised in the outer mitochondrial membrane. Studies have demonstrated knockout of USP30 to enhance mitophagy in both cellular and animal models. [21,25,28,29]. In PINK1 or Parkin knockout *Drosophila* models, USP30 knockout was able to protect from a PD-like motor phenotype [25].

Initially, we confirmed that siRNA-mediated knockdown of USP30 increases stress-induced mitophagy. We utilised the neuroblastoma SHSY5Y cell line and showed that knockdown of USP30 could be demonstrated to enhance the mitoKeima signal, indicating changes in the mitophagy pathway, in agreement with the other studies [25]. Knockdown of USP30, however, seems to affect the function of the mitochondrial ETC since a decrease in basal respiration and oxygen used for ATP synthesis was determined which was also accompanied by an increase in the mitochondrial membrane potential. To generate ATP, mitochondria utilise the proton electrochemical gradient potential generated by serial reductions of electrons in the ETC. The reductive transfer of electrons through the ETC complexes provides the force to drive protons from the mitochondrial matrix to outer face of the inner mitochondrial membrane against their concentration gradient. The accumulated protons in the inter membrane space then flow back into the mitochondrial matrix through Complex V (ATP synthase), producing ATP. These reactions result in the formation of a voltage and a pH gradient [53]. Studies have shown that increased mitochondrial membrane potential can be caused by inhibition of Complex I and Complex V of the ETC [54,55]. Inhibition of these complexes could then lead to reduced OCR levels. The effect of USP30 knockdown in the OCR and inner mitochondrial membrane potential, assuming no off-target effects could possibly be attributed to the newly characterised role of USP30 in regulating protein import in the mitochondria [30,31]. Phu *et al*. [31] has recently shown that a number of proteins belonging to the ETC (such as ATPB, NDUA6 and COX4-1) presented increased ubiquitination when USP30 was inhibited. The absence of these proteins from the inner mitochondrial membrane could result in a compromised ETC that could lead to reduced respiration. Another study by Gu *et al*. [34] has reported reduced OCR in primary hepatocytes from USP30 knockout mice where they identified that USP30 can be phosphorylated by IKKβ, resulting in deubiquitination of ATP citrate lyase and fatty acid synthase. Both our results and the study from Gu *et al*. indicate that USP30 could potentially regulate different metabolic pathways such as ATP production and lipogenesis.

The observed results with the genetic perturbation of USP30 were replicated with the small molecule inhibitor USP30Inh-1 resulting in a dose-dependent increase in the mitoKeima signal when mitophagy was induced with A/O. However, in the SHSY5Y cells, 10 µM USP30Inh-1 caused a depolarisation of the mitochondrial inner membrane potential when cells were treated for 1 h. Prolonged incubation seems to restore the mitochondrial membrane potential whereas lower concentrations (0.1 µM) do not seem to affect it. A small but significant decrease in TMRM was also observed in the DA neurones/astrocytes co-cultures when treated with 3 µM USP30Inh-1 for 4 days. The acute drop in the TMRM signal after 1 h incubation with 10 μM USP30Inh-1 could mean that the cells were metabolically affected by pharmacological inhibition of USP30 and/or other targets of USP30Inh-1. Based on the DUB profiling treatment with 10 μM USP30Inh-1, where we see the highest toxicity, seems to target other DUBs (e.g. USP21 and USP45), which indicates that the compound could potentially have other intracellular targets. A significant decrease in mitochondrial potential could indicate that at higher concentration USP30Inh-1 is acutely toxic to cells by possibly affecting the enzymes of the ETC that are responsible for the maintenance of the inner membrane potential, in line with a suggested role for USP30 in mitochondrial protein import [30]. For transient effects on TMRM, a compensation mechanism may take place to result in partial restoration of the mitochondrial inner membrane potential. Nonetheless, we are able to show that a non-toxic concentration (0.1 µM) could also increase the mitoKeima signal when A/O was used to induced mitophagy.

The USP30 inhibitors used here are proposed to form an adduct between the cyano-amide group and the cysteine residue in the active side of the protein. Recently, two other USP30 inhibitors were reported containing the cyano-amide group. Phu *et al*. [31] reported USP30i which was able to increase TOM20 ubiquitination at concentrations above 5 µM in HEK293 cells overexpressing Parkin. The second study comes from Rusilowicz-Jones *et al*. [37], where they characterised the compound FT3967385 which increased TOM20 ubiquitination, mitolysosomal formation via monitoring the mitoQC signal and p-Ser65-Ub levels at 200 nM in SHSY5Y cells. There has only been limited assessment of overall mitochondrial function for these compounds, and our study suggests that careful evaluation of effects on mitochondrial health is important when considering applications in cell biology. Equally, compounds such as USP30Inh-1 have not been completely characterised and it is possible that they have effects and targets beyond those explored in this study.

The above-mentioned inhibitors inactivate USP30 by forming a covalent bond with the cysteine in the active site of USP30. Compounds that contain the racemic phenylalanine group, which form non-covalent bond with USP30, were also reported [56–58]. Kluge *et al*. [56] initially reported another compound, MF-094, as a highly selective USP30 inhibitor that was able to increase mitophagy in C2C12 myotubes when measuring mtDNA levels. Luo *et al*. [57] tested a compound called ST-593 in both a cellular and a mouse model. ST-593 increased TOM20 ubiquitination and mitoKeima signal without affecting the mitochondrial membrane potential in HeLa cells overexpressing Parkin. Furthermore, they observed increased mitophagy in heart tissue of mice but not in liver tissue after treatment with ST-593 [57]. Rusilowicz-Jones *et al*. [58] used an USP30 inhibitor named “Compound 39” and reported increased TOM20 ubiquitination, p-Ser65-Ub and mitophagy in SHSY5Y cells. Compound 39 was also tested in dopaminergic neurones carrying loss of function mutations of Parkin which was able to restore mitophagy almost back to control levels [58]. The above-mentioned studies were able to show increase mitophagy in different cellular models upon pharmacological inhibition of USP30. However, the mouse study indicates that mitophagy might differ amongst tissues [57]. Future studies where all these USP30 inhibitors are tested under the same disease-relevant model will probably provide a better understanding on their function and usage as potential drugs targeting USP30.

Furthermore, we evaluated genetic and pharmacological inhibition of USP30 in a PD-relevant model. Following mitochondrial damage, both Parkin and ubiquitin are phosphorylated by PINK1 on Ser65 residues [59,60]. These phosphorylation events result in full activation of Parkin E3-ligase activity and translocation to mitochondria, tagging them for removal by the autophagosome-lysosome system [47,61,62]. The R275W mutation within the RING1 domain of Parkin is one of the most common autosomal recessive, early onset-associated variants [63]. The R275W mutation has a limited effect on Parkin recruitment to depolarised mitochondria but causes severe disruption of subsequent mitochondrial clearance [38,64]. Interestingly, a low level of mono-ubiquitinated VDAC1 has been detected with Parkin R275W, suggesting remaining, residual enzymatic functionality [64]. These observations are consistent with small depolarisation-induced increases in Mfn2-Ub and p-Ser65-Ub in the Parkin^R275W/R275Q^ fibroblasts (Figure 7).

Parkin is composed of an N-terminal Ubl domain and four RING-like domains (RING0, RING1, IBR and RING2). Upon activation, conformational change results in rearrangements of these domains to allow coordination of the p-Ser65-Ub molecule. The Ubl domain interacts with helix 261–274 within the RING1 region, served by hydrogen bonds between Asp274 immediately preceding the Arg275 mutation site [65]. The Arg275 mutation site interacts with Glu321 in the p-Ser65-Ub-binding helix, and the R275W mutation has been proposed to cause clashes within the p-Ser65-Ub-binding helix and with regions of the Ubl domain [38]. Together, these structural insights predict the Arg275 mutation to lead to Parkin destabilisation, and are consistent with observations of reduced Parkin protein expression and cellular aggregation [38,66]. Here, we have characterised patient-derived familial PD fibroblasts with verified R275 mutation in Parkin. In agreement with structural and functional insights, heterozygous and compound heterozygous mutant fibroblasts demonstrate: mitophagy deficits; reduced Mfn-2 ubiquitination and clearance; and decreased p-Ser65-Ub, with a clear genotype-phenotype relationship determined by allelic number of *PRKN* R275 mutations. Furthermore, re-introduction of common variant Parkin protein can rescue mono- and bi-allelic mutation at R275, restoring p-Ser65-Ub levels to that of control fibroblasts.

Considering both the proposed role of USP30 in controlling initiation of mitophagy and the structural and functional elements of the Parkin R275W mutation, we focused upstream within the PINK1/Parkin cascade to explore and identify biochemical changes associated with genetic and small molecule USP30 inhibition in this context. To date, PINK1 is the only recognised ubiquitin kinase [60,67]; consequently, Ser65 phosphorylation of ubiquitin is a key biomarker of cellular efficacy in recognition and clearance of dysfunctional mitochondria. Following mitochondrial uncoupling, small molecule USP30 inhibition was able, under appropriate conditions, to rescue Parkin-dependent deficits in mitophagy signalling, restoring p-Ser65-Ub and ubiquitinated Mfn2 toward Parkin^+/+^ levels. Notably, this phenotype was clearly observed in Parkin^+/R275W^ fibroblasts but not with the Parkin^R275W/R275Q^. Equally, expression of a catalytically dead version of USP30 was able to phenocopy pharmacological USP30 inhibition with respect to increasing mitochondrial p-Ser65-Ub. These data strongly suggest a requirement for functional Parkin in mediating USP30-dependent ubiquitination dynamics.

In this report, we have explored pharmacological and genetic inhibition of USP30 in multiple cell models. We have: (1) demonstrated that the PINK1–Parkin–USP30 axis is conserved across cell types (DA neurones, astrocytes, SHSY5Y and fibroblasts); (2) expanded the USP30 inhibitor toolbox by characterising an additional small molecule inhibitor of USP30 that increases the mitoKeima signal in SHSY5Y cells when using non-toxic concentrations, consistent with the effects of genetic experimental perturbation of USP30; (3) characterised a panel of patient-derived fibroblasts carrying Parkin mutations, demonstrating defects in Parkin function; (4) recognised that genetic or pharmacological USP30 inhibition can rescue Parkin-dependent signalling defects and (5), shown that Parkin activity is necessary for USP30 inhibitor-driven events during the early steps of mitophagy initiation.

Our findings indicate that USP30 may have a significant role in regulating Parkin-mediated mitophagy as well as ubiquitination and the PINK1-dependent phosphorylation of ubiquitin. Genetic inhibition of USP30 was able to rescue Parkin-dependent deficits in the mitophagy pathway. However, USP30 seems also to have an important role in the ETC function since its genetic inhibition caused metabolic changes. The pharmacologic inhibition of USP30 presented off-target effects as well as increased toxicity at the highest tested concentrations indicating that more specific and possibly chemical diverse compounds are required. Further studies will be required to explore the role of USP30 in mitochondrial function and metabolic homeostasis.

## Data Availability

All data that support the findings of this study are included in this manuscript. Further information is available upon request.

## Open Access Statement

Open access for this article was enabled by the participation of University College London in an all-inclusive Read & Publish pilot with Portland Press and the Biochemical Society under a transformative agreement with JISC.

## Abbreviations

ABP: activity-based probe
DUB: de-ubiquitinating
ETC: electron transport chain
GFAP: glial fibrillary acidic protein
OCR: oxygen consumption rate
PD: Parkinson disease
PINK1: PTEN-induced kinase 1
TOM20: translocase of outer mitochondrial membrane 20
TUBEs: Tandem Ubiquitin Binding Entities

## References

[1] Pickles, S., Vigié, P. and Youle, R.J. (2018) Mitophagy and quality control mechanisms in mitochondrial maintenance. Curr. Biol. 28, R170–R185 10.1016/j.cub.2018.01.004 29462587 PMC7255410

[2] Calkins, M.J., Manczak, M., Mao, P., Shirendeb, U. and Reddy, P.H. (2011) Impaired mitochondrial biogenesis, defective axonal transport of mitochondria, abnormal mitochondrial dynamics and synaptic degeneration in a mouse model of Alzheimer's disease. Hum. Mol. Genet. 20, 4515–4529 10.1093/hmg/ddr381 21873260 PMC3209824

[3] Rodolfo, C., Campello, S. and Cecconi, F. (2018) Mitophagy in neurodegenerative diseases. Neurochem. Int. 117, 156–166 10.1016/j.neuint.2017.08.004 28797885

[4] Clark, E.H., Vázquez De La Torre, A., Hoshikawa, T. and Briston, T. (2021) Targeting mitophagy in Parkinson's disease. J. Biol. Chem. 296, 100209 10.1074/jbc.REV120.014294 33372898 PMC7948953

[5] Malpartida, A.B., Williamson, M., Narendra, D.P., Wade-Martins, R. and Ryan, B.J. (2021) Mitochondrial dysfunction and mitophagy in Parkinson's disease: from mechanism to therapy. Trends Biochem. Sci. 46, 329–343 10.1016/j.tibs.2020.11.007 33323315

[6] Grünewald, A., Kumar, K.R. and Sue, C.M. (2019) New insights into the complex role of mitochondria in Parkinson's disease. Prog. Neurobiol. 177, 73–93 10.1016/j.pneurobio.2018.09.003 30219247

[7] Schapira, A.H.V., Cooper, J.M., Dexter, D., Jenner, P., Clark, J.B. and Marsden, C.D. (1989) Mitochondrial complex I deficiency in Parkinson's disease. Lancet 333, 1269 10.1016/S0140-6736(89)92366-0 2566813

[8] Bender, A., Krishnan, K.J., Morris, C.M., Taylor, G.A., Reeve, A.K., Perry, R.H. et al. (2006) High levels of mitochondrial DNA deletions in substantia nigra neurons in aging and Parkinson disease. Nat. Genet. 38, 515–517 10.1038/ng1769 16604074

[9] Zarow, C., Lyness, S.A., Mortimer, J.A. and Chui, H.C. (2003) Neuronal loss is greater in the locus coeruleus than nucleus basalis and substantia nigra in Alzheimer and Parkinson diseases. Arch. Neurol. 60, 337–341 10.1001/archneur.60.3.337 12633144

[10] Miller, S. and Muqit, M.M.K. (2019) Therapeutic approaches to enhance PINK1/Parkin mediated mitophagy for the treatment of Parkinson's disease. Neurosci. Lett. 705, 7–13 10.1016/j.neulet.2019.04.029 30995519

[11] Moehlman, A.T. and Youle, R.J. (2020) Mitochondrial quality control and restraining innate immunity. Annu. Rev. Cell Dev. Biol. 36, 265–289 10.1146/annurev-cellbio-021820-101354 33021820

[12] Kitada, T., Asakawa, S., Hattori, N., Matsumine, H., Yamamura, Y., Minoshima, S. et al. (1998) Mutations in the parkin gene cause autosomal recessive juvenile parkinsonism. Nature 392, 605–608 10.1038/33416 9560156

[13] Valente, E.M., Abou-Sleiman, P.M., Caputo, V., Muqit, M.M., Harvey, K., Gispert, S. et al. (2004) Hereditary early-onset Parkinson's disease caused by mutations in PINK1. Science 304, 1158–1160 10.1126/science.1096284 15087508

[14] McWilliams, T.G., Barini, E., Pohjolan-Pirhonen, R., Brooks, S.P., Singh, F., Burel, S. et al. (2018) Phosphorylation of Parkin at serine 65 is essential for its activation in vivo. Open Biol. 8, 180108 10.1098/rsob.180108 30404819 PMC6282074

[15] Sarraf, S.A., Raman, M., Guarani-Pereira, V., Sowa, M.E., Huttlin, E.L., Gygi, S.P. et al. (2013) Landscape of the PARKIN-dependent ubiquitylome in response to mitochondrial depolarization. Nature 496, 372–376 10.1038/nature12043 23503661 PMC3641819

[16] Lazarou, M., Sliter, D.A., Kane, L.A., Sarraf, S.A., Wang, C., Burman, J.L. et al. (2015) The ubiquitin kinase PINK1 recruits autophagy receptors to induce mitophagy. Nature 524, 309–314 10.1038/nature14893 26266977 PMC5018156

[17] Durcan, T.M. and Fon, E.A. (2015) The three ‘P's of mitophagy: PARKIN, PINK1, and post-translational modifications. Genes Dev. 29, 989–999 10.1101/gad.262758.115 25995186 PMC4441056

[18] Sun, N., Youle, R.J. and Finkel, T. (2016) The mitochondrial basis of aging. Mol. Cell 61, 654–666 10.1016/j.molcel.2016.01.028 26942670 PMC4779179

[19] Stead, E.R., Castillo-Quan, J.I., Miguel, V.E.M., Lujan, C., Ketteler, R., Kinghorn, K.J. et al. (2019) Agephagy: adapting autophagy for health during aging. Front. Cell Dev. Biol. 7, 308 10.3389/fcell.2019.00308 31850344 PMC6892982

[20] Singh, P.K. and Muqit, M.M.K. (2020) Parkinson's: a disease of aberrant vesicle trafficking. Annu. Rev. Cell Dev. Biol. 36, 237–264 10.1146/annurev-cellbio-100818-125512 32749865

[21] Padmanabhan, S., Polinski, N.K., Menalled, L.B., Baptista, M.A.S. and Fiske, B.K. (2019) The Michael J. Fox foundation for Parkinson's research strategy to advance therapeutic development of PINK1 and Parkin. Biomolecules 9, 296 10.3390/biom9080296 PMC6723155 31344817

[22] Clague, M.J., Urbé, S. and Komander, D. (2019) Breaking the chains: deubiquitylating enzyme specificity begets function. Nat. Rev. Mol. Cell Biol. 20, 338–352 10.1038/s41580-019-0099-1 30733604

[23] Durcan, T.M., Tang, M.Y., Pérusse, J.R., Dashti, E.A., Aguileta, M.A., McLelland, G.L. et al. (2014) USP 8 regulates mitophagy by removing K 6-linked ubiquitin conjugates from parkin. EMBO J. 33, 2473–2491 10.15252/embj.201489729 25216678 PMC4283406

[24] Cornelissen, T., Haddad, D., Wauters, F., Van Humbeeck, C., Mandemakers, W., Koentjoro, B. et al. (2014) The deubiquitinase USP15 antagonizes Parkin-mediated mitochondrial ubiquitination and mitophagy. Hum. Mol. Genet. 23, 5227–5242 10.1093/hmg/ddu244 24852371 PMC7108632

[25] Bingol, B., Tea, J.S., Phu, L., Reichelt, M., Bakalarski, C.E., Song, Q. et al. (2014) The mitochondrial deubiquitinase USP30 opposes parkin-mediated mitophagy. Nature 510, 370–375 10.1038/nature13418 24896179

[26] Niu, K., Fang, H., Chen, Z., Zhu, Y., Tan, Q., Wei, D. et al. (2020) USP33 deubiquitinates PRKN/parkin and antagonizes its role in mitophagy. Autophagy 16, 724–734 10.1080/15548627.2019.1656957 31432739 PMC7138199

[27] Wang, Y., Serricchio, M., Jauregui, M., Shanbhag, R., Stoltz, T., Di Paolo, C.T. et al. (2015) Deubiquitinating enzymes regulate PARK2-mediated mitophagy. Autophagy 11, 595–606 10.1080/15548627.2015.1034408 25915564 PMC4502823

[28] Liang, J.R., Martinez, A., Lane, J.D., Mayor, U., Clague, M.J. and Urbe, S. (2015) USP30 deubiquitylates mitochondrial Parkin substrates and restricts apoptotic cell death. EMBO Rep. 16, 618–627 10.15252/embr.201439820 25739811 PMC4428036

[29] Marcassa, E., Kallinos, A., Jardine, J., Rusilowicz-Jones, E.V., Martinez, A., Kuehl, S. et al. (2018) Dual role of USP 30 in controlling basal pexophagy and mitophagy. EMBO Rep. 19, e45595 10.15252/embr.201745595 29895712 PMC6030704

[30] Ordureau, A., Paulo, J.A., Zhang, J., An, H., Swatek, K.N., Cannon, J.R. et al. (2020) Global landscape and dynamics of Parkin and USP30-dependent ubiquitylomes in iNeurons during mitophagic signaling. Mol. Cell 77, 1124–1142 e1110 10.1016/j.molcel.2019.11.013 32142685 PMC7098486

[31] Phu, L., Rose, C.M., Tea, J.S., Wall, C.E., Verschueren, E., Cheung, T.K. et al. (2020) Dynamic regulation of mitochondrial import by the ubiquitin system. Mol. Cell 77, 1107–1123.e1110 10.1016/j.molcel.2020.02.012 32142684

[32] Soutar, M.P.M., Kempthorne, L., Annuario, E., Luft, C., Wray, S., Ketteler, R. et al. (2019) FBS/BSA media concentration determines CCCP's ability to depolarize mitochondria and activate PINK1-PRKN mitophagy. Autophagy 15, 2002–2011 10.1080/15548627.2019.1603549 31060423 PMC6844515

[33] Katayama, H., Kogure, T., Mizushima, N., Yoshimori, T. and Miyawaki, A. (2011) A sensitive and quantitative technique for detecting autophagic events based on lysosomal delivery. Chem. Biol. 18, 1042–1052 10.1016/j.chembiol.2011.05.013 21867919

[34] Gu, L., Zhu, Y., Lin, X., Lu, B., Zhou, X., Zhou, F. et al. (2020) The IKKβ-USP30-ACLY axis controls lipogenesis and tumorigenesis. Hepatology 73, 160–174 10.1002/hep.31249 32221968

[35] Sato, Y., Okatsu, K., Saeki, Y., Yamano, K., Matsuda, N., Kaiho, A. et al. (2017) Structural basis for specific cleavage of Lys6-linked polyubiquitin chains by USP30. Nat. Struct. Mol. Biol. 24, 911–919 10.1038/nsmb.3469 28945247

[36] Gersch, M., Gladkova, C., Schubert, A.F., Michel, M.A., Maslen, S. and Komander, D. (2017) Mechanism and regulation of the Lys6-selective deubiquitinase USP30. Nat. Struct. Mol. Biol. 24, 920–930 10.1038/nsmb.3475 28945249 PMC5757785

[37] Rusilowicz-Jones, E.V., Jardine, J., Kallinos, A., Pinto-Fernandez, A., Guenther, F., Giurrandino, M. et al. (2020) USP30 sets a trigger threshold for PINK1–PARKIN amplification of mitochondrial ubiquitylation. Life Sci. Alliance 3, e202000768 10.26508/lsa.202000768 32636217 PMC7362391

[38] Yi, W., MacDougall, E.J., Tang, M.Y., Krahn, A.I., Gan-Or, Z., Trempe, J.F. et al. (2019) The landscape of Parkin variants reveals pathogenic mechanisms and therapeutic targets in Parkinson's disease. Hum. Mol. Genet. 28, 2811–2825 10.1093/hmg/ddz080 30994895 PMC6736174

[39] Poole, A.C., Thomas, R.E., Yu, S., Vincow, E.S. and Pallanck, L. (2010) The mitochondrial fusion-promoting factor mitofusin is a substrate of the PINK1/Parkin pathway. PLoS ONE 5, e10054 10.1371/journal.pone.0010054 20383334 PMC2850930

[40] Gegg, M.E., Cooper, J.M., Chau, K.-Y., Rojo, M., Schapira, A.H.V. and Taanman, J.-W. (2010) Mitofusin 1 and mitofusin 2 are ubiquitinated in a PINK1/parkin-dependent manner upon induction of mitophagy. Hum. Mol. Genet. 19, 4861–4870 10.1093/hmg/ddq419 20871098 PMC3583518

[41] Chan, N.C., Salazar, A.M., Pham, A.H., Sweredoski, M.J., Kolawa, N.J., Graham, R.L.J. et al. (2011) Broad activation of the ubiquitin–proteasome system by Parkin is critical for mitophagy. Hum. Mol. Genet. 20, 1726–1737 10.1093/hmg/ddr048 21296869 PMC3071670

[42] Lopitz-Otsoa, F., Rodriguez-Suarez, E., Aillet, F., Casado-Vela, J., Lang, V., Matthiesen, R. et al. (2012) Integrative analysis of the ubiquitin proteome isolated using tandem ubiquitin binding entities (TUBEs). J. Proteomics 75, 2998–3014 10.1016/j.jprot.2011.12.001 22178446

[43] Bose, A. and Beal, M.F. (2016) Mitochondrial dysfunction in Parkinson's disease. J. Neurochem. 139, 216–231 10.1111/jnc.13731 27546335

[44] Hasson, S.A., Kane, L.A., Yamano, K., Huang, C.H., Sliter, D.A., Buehler, E. et al. (2013) High-content genome-wide RNAi screens identify regulators of parkin upstream of mitophagy. Nature 504, 291–295 10.1038/nature12748 24270810 PMC5841086

[45] Park, J., Lee, S.B., Lee, S., Kim, Y., Song, S., Kim, S. et al. (2006) Mitochondrial dysfunction in drosophila PINK1 mutants is complemented by parkin. Nature 441, 1157–1161 10.1038/nature04788 16672980

[46] Clark, I.E. Dodson, M.W., Jiang, C., Cao, J.H., Huh, J.R., Seol, J.H. et al. (2006) Drosophila pink1 is required for mitochondrial function and interacts genetically with parkin. Nature 441, 1162–1166 10.1038/nature04779 16672981

[47] Narendra, D., Tanaka, A., Suen, D.F. and Youle, R.J. (2008) Parkin is recruited selectively to impaired mitochondria and promotes their autophagy. J. Cell Biol. 183, 795–803 10.1083/jcb.200809125 19029340 PMC2592826

[48] Hsieh, C.H., Shaltouki, A., Gonzalez, A.E., Bettencourt da Cruz, A., Burbulla, L.F., St Lawrence, E. et al. (2016) Functional impairment in miro degradation and mitophagy is a shared feature in familial and sporadic Parkinson's disease. Cell Stem Cell 19, 709–724 10.1016/j.stem.2016.08.002 27618216 PMC5135570

[49] Ambrosi, G., Ghezzi, C., Sepe, S., Milanese, C., Payan-Gomez, C., Bombardieri, C.R. et al. (2014) Bioenergetic and proteolytic defects in fibroblasts from patients with sporadic Parkinson's disease. Biochim. Biophys. Acta 1842, 1385–1394 10.1016/j.bbadis.2014.05.008 24854107

[50] Smith, G.A., Jansson, J., Rocha, E.M., Osborn, T., Hallett, P.J. and Isacson, O. (2016) Fibroblast biomarkers of sporadic Parkinson's disease and LRRK2 kinase inhibition. Mol. Neurobiol. 53, 5161–5177 10.1007/s12035-015-9435-4 26399642 PMC5012155

[51] Grünewald, A., Voges, L., Rakovic, A., Kasten, M., Vandebona, H., Hemmelmann, C. et al. (2010) Mutant Parkin impairs mitochondrial function and morphology in human fibroblasts. PLoS ONE 5, e12962 10.1371/journal.pone.0012962 20885945 PMC2946349

[52] Langley, M., Ghosh, A., Charli, A., Sarkar, S., Ay, M., Luo, J. et al. (2017) Mito-apocynin prevents mitochondrial dysfunction, microglial activation, oxidative damage, and progressive neurodegeneration in mitopark transgenic mice. Antioxid. Redox Signal. 27, 1048–1066 10.1089/ars.2016.6905 28375739 PMC5651937

[53] Mitchell, P. (1966) Chemiosmotic coupling in oxidative and photosynthetic phosphorylation. Biol. Rev. 41, 445–502 10.1111/j.1469-185X.1966.tb01501.x 5329743

[54] Forkink, M., Manjeri, G.R., Liemburg-Apers, D.C., Nibbeling, E., Blanchard, M., Wojtala, A. et al. (2014) Mitochondrial hyperpolarization during chronic complex I inhibition is sustained by low activity of complex II, III, IV and V. Biochim. Biophys. Acta Bioenergetics 1837, 1247–1256 10.1016/j.bbabio.2014.04.008 24769419

[55] Solaini, G., Sgarbi, G., Lenaz, G. and Baracca, A. (2007) Evaluating mitochondrial membrane potential in cells. Biosci. Rep. 27, 11–21 10.1007/s10540-007-9033-4 17497220

[56] Kluge, A.F., Lagu, B.R., Maiti, P., Jaleel, M., Webb, M., Malhotra, J. et al. (2018) Novel highly selective inhibitors of ubiquitin specific protease 30 (USP30) accelerate mitophagy. Bioorg. Med. Chem. Lett. 28, 2655–2659 10.1016/j.bmcl.2018.05.013 29935771

[57] Luo, H., Krigman, J., Zhang, R., Yang, M. and Sun, N. (2021) Pharmacological inhibition of USP30 activates tissue-specific mitophagy. Acta Physiol. 232, e13666 10.1111/apha.13666 PMC8266733 33890401

[58] Rusilowicz-Jones, E.V., Barone, F.G., Lopes, F.M., Stephen, E., Mortiboys, H., Urbé, S. et al. (2021) Benchmarking A Highly Selective USP30 Inhibitor for Enhancement of Mitophagy and Pexophagy, Cold Spring Harbor Laboratory 10.26508/lsa.202101287 PMC8645336 34844982

[59] Kondapalli, C., Kazlauskaite, A., Zhang, N., Woodroof, H.I., Campbell, D.G., Gourlay, R. et al. (2012) PINK1 is activated by mitochondrial membrane potential depolarization and stimulates Parkin E3 ligase activity by phosphorylating serine 65. Open Biol. 2, 120080 10.1098/rsob.120080 22724072 PMC3376738

[60] Kane, L.A., Lazarou, M., Fogel, A.I., Li, Y., Yamano, K., Sarraf, S.A. et al. (2014) PINK1 phosphorylates ubiquitin to activate Parkin E3 ubiquitin ligase activity. J. Cell Biol. 205, 143–153 10.1083/jcb.201402104 24751536 PMC4003245

[61] Wauer, T., Simicek, M., Schubert, A. and Komander, D. (2015) Mechanism of phospho-ubiquitin-induced PARKIN activation. Nature 524, 370–374 10.1038/nature14879 26161729 PMC4984986

[62] Kazlauskaite, A., Martinez-Torres, R.J., Wilkie, S., Kumar, A., Peltier, J., Gonzalez, A. et al. (2015) Binding to serine 65-phosphorylated ubiquitin primes Parkin for optimal PINK1-dependent phosphorylation and activation. EMBO Rep. 16, 939–954 10.15252/embr.201540352 26116755 PMC4552487

[63] Bayne, A.N. and Trempe, J.-F. (2019) Mechanisms of PINK1, ubiquitin and Parkin interactions in mitochondrial quality control and beyond. Cell. Mol. Life Sci. 76, 4589–4611 10.1007/s00018-019-03203-4 31254044 PMC11105328

[64] Geisler, S., Holmström, K.M., Skujat, D., Fiesel, F.C., Rothfuss, O.C., Kahle, P.J. et al. (2010) PINK1/Parkin-mediated mitophagy is dependent on VDAC1 and p62/SQSTM1. Nat. Cell Biol. 12, 119–131 10.1038/ncb2012 20098416

[65] Sauvé, V., Lilov, A., Seirafi, M., Vranas, M., Rasool, S., Kozlov, G. et al. (2015) A Ubl/ubiquitin switch in the activation of Parkin. EMBO J. 34, 2492–2505 10.15252/embj.201592237 26254305 PMC4609182

[66] Hampe, C., Ardila-Osorio, H., Fournier, M., Brice, A. and Corti, O. (2006) Biochemical analysis of Parkinson's disease-causing variants of Parkin, an E3 ubiquitin–protein ligase with monoubiquitylation capacity. Hum. Mol. Genet. 15, 2059–2075 10.1093/hmg/ddl131 16714300

[67] Schubert, A.F., Gladkova, C., Pardon, E., Wagstaff, J.L., Freund, S.M.V., Steyaert, J. et al. (2017) Structure of PINK1 in complex with its substrate ubiquitin. Nature 552, 51 10.1038/nature24645 29160309 PMC6020998

