# Supplementary material for: Investigation of USP30 inhibition to enhance Parkin-mediated mitophagy: tools and approaches
Source: Biochem J. 2021 Dec 6;478(23):4099–118. doi: 10.1042/BCJ20210508 (PMC8718267; doi:10.1042/BCJ20210508)
Supplement: Supplementary Figures S1-S5 [file BCJ-478-4099-s1.pdf]

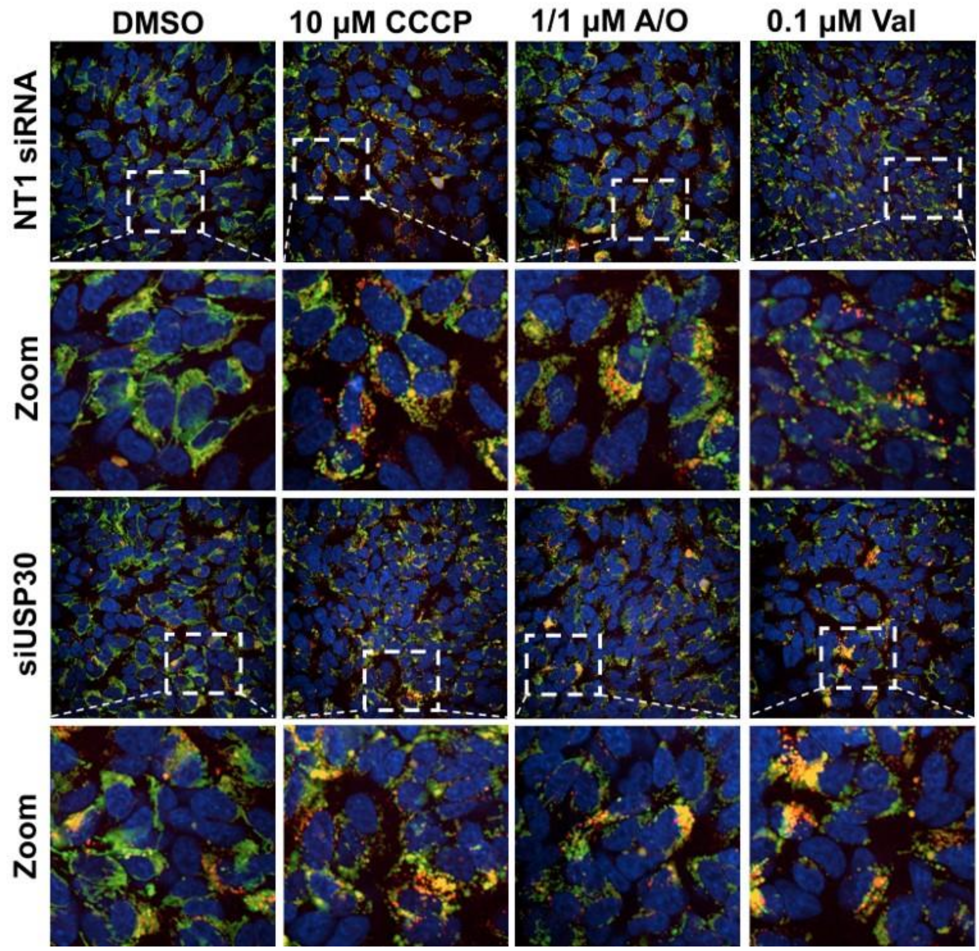

Supplementary Figure S1. Changes of the mitoKeima signal after siRNA-mediated knockdown of USP30.

Representative images of USP30 knockdown in SHSY5Y mitoKeima cells for 7 days before inducing mitophagy with 10 µM CCCP or 1/1 µM A/O or 0.1 µM Val for 7 h.

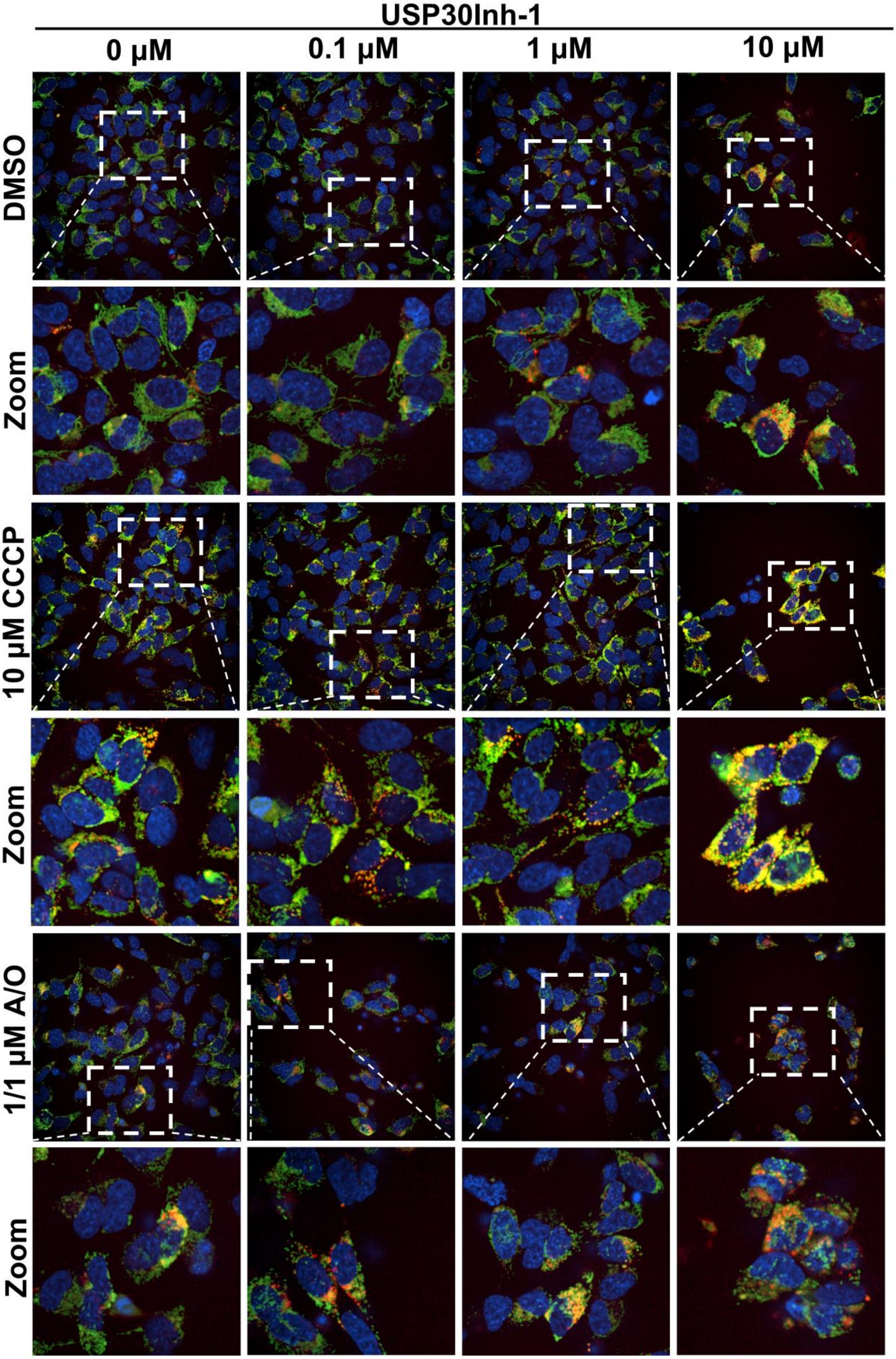
USP30Inh-1
0 µM
0.1 µM
1 µM
10 µM
DMSO
Zoom
10 µM CCCP
Zoom
1/1 µM A/O
Zoom

Supplementary Figure S2. Changes of the mitoKeima signal after treatment with USP30Inh-1 for 3 days.

Representative images of SHSY5Y mitoKeima cells treated with the indicated USP30Inh-1 concentration for 3 days. Mitophagy was induced with 1/1 μM A/O or 10 μM CCCP for 10 h.

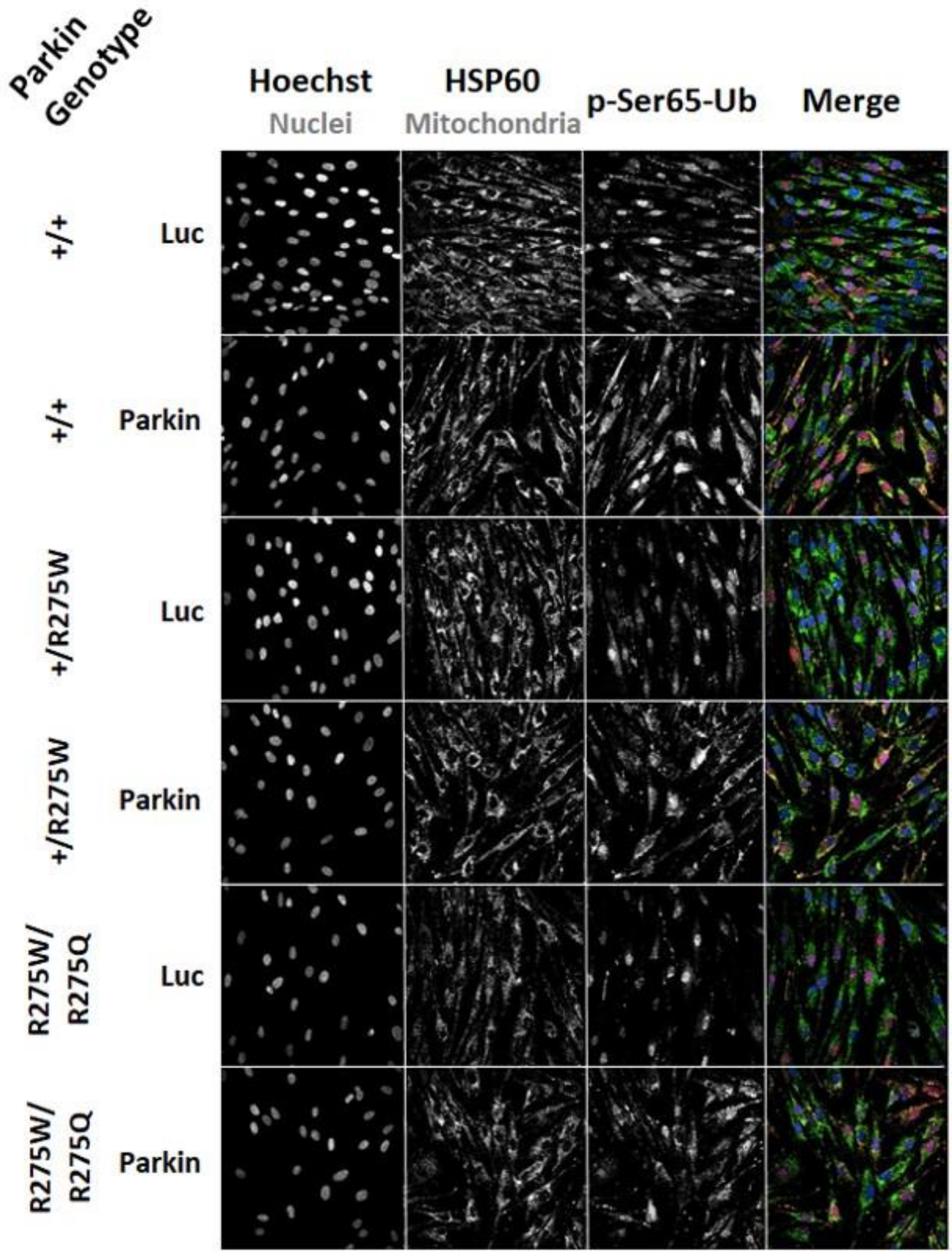

Supplementary Figure S3. Over-expression of WT Parkin in patient-derived Parkin mutated fibroblasts.

Parkin$^{+/+}$, Parkin$^{+/R275W}$ and Parkin$^{R275W/R275Q}$ fibroblasts were transfected with a Parkin WT mRNA or Luciferase (Luc) mRNA for 20 h before inducing mitophagy for 2 h with 10 µM FCCP. Representative images of the immuno-staining for p-Ser65-Ub and HSP60 are presented.

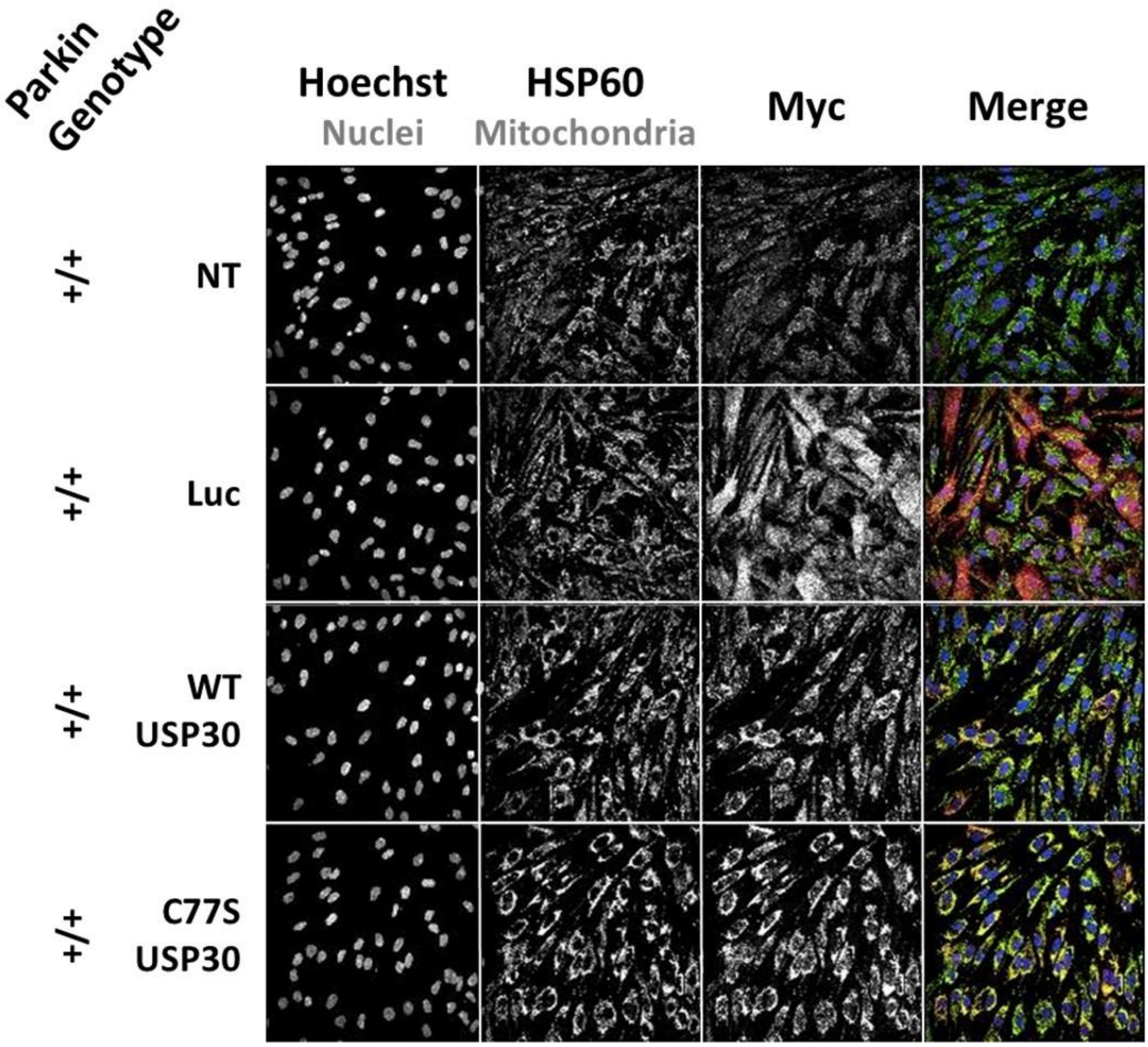

Supplementary Figure S4. Over-expression of USP30-C77S in Parkin$^{+/+}$ fibroblasts.

Representative images of Parkin$^{+/+}$ fibroblasts transfected with a non-targeted (NT), Luciferase (Luc), USP30-WT or USP30-C77S mRNA for 20 h. Transfection efficiency was assessed via immuno-staining for Myc (epitope tag for USP30) and HSP60 as mitochondria marker.

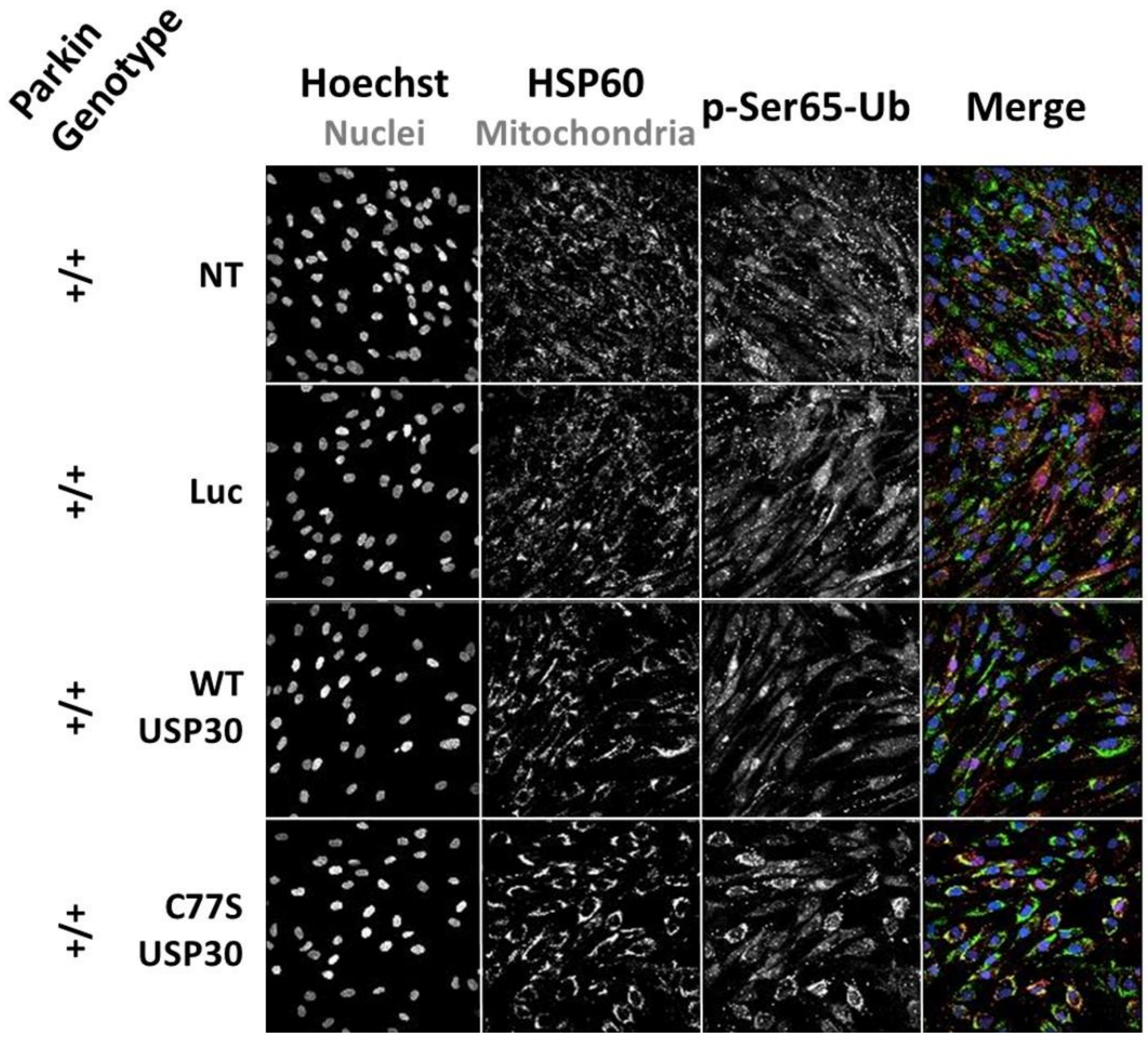

Supplementary Figure S5. Over-expression of USP30-C77S affects the p-Ser65-Ub levels in Parkin$^{+/+}$ fibroblasts.

The Parkin$^{+/+}$ fibroblasts were transfected with non-targeted (NT), Luciferase (Luc), USP30-WT or USP30-C77S mRNA for 20 h before inducing mitophagy for 2 h with 10 μM FCCP. Representative images of the immuno-staining for p-Ser65-Ub and HSP60 are presented.
